# Widespread adaptive evolution in angiosperm photosystems provides insight into the evolution of photosystem II repair

**DOI:** 10.1093/plcell/koae281

**Published:** 2024-10-15

**Authors:** Elizabeth H J Robbins, Steven Kelly

**Affiliations:** Department of Biology, University of Oxford, South Parks Road, Oxford OX1 3RB, UK; Department of Biology, University of Oxford, South Parks Road, Oxford OX1 3RB, UK

## Abstract

Oxygenic photosynthesis generates the initial energy source that fuels nearly all life on Earth. At the heart of the process are the photosystems, which are pigment binding multiprotein complexes that catalyze the first step of photochemical conversion of light energy into chemical energy. Here, we investigate the molecular evolution of the plastid-encoded photosystem subunits at single-residue resolution across 773 angiosperm species. We show that despite an extremely high level of conservation, 7% of residues in the photosystems, spanning all photosystem subunits, exhibit hallmarks of adaptive evolution. Through *in silico* modeling of these adaptive substitutions, we uncover the impact of these changes on the predicted properties of the photosystems, focusing on their effects on cofactor binding and intersubunit interface formation. By analyzing these cohorts of changes, we reveal that evolution has repeatedly altered the interaction between Photosystem II and its D1 subunit in a manner that is predicted to reduce the energetic barrier for D1 turnover and photosystem repair. Together, these results provide insight into the trajectory of photosystem adaptation during angiosperm evolution.

## Teaser

Extensive adaptive evolution in angiosperm photosystems provides insight into the past, present, and future evolution of photosynthesis in plants.

## Introduction

Oxygenic photosynthesis is the process that converts sunlight and carbon dioxide into sugars and oxygen and fuels nearly all life on earth. Although it remains unclear exactly when oxygenic photosynthesis first arose, there is evidence to suggest it had already evolved by ∼3 billion years ago (Planavsky et al. 2014; Fournier et al. 2021), and subsequently facilitated the rise of molecular oxygen in the atmosphere and the evolution of aerobic organisms (Eigenbrode and Freeman 2006; Koch and Britton 2008). At the heart of oxygenic photosynthesis are the two photosystem complexes, Photosystem I (PSI) and Photosystem II (PSII) (Blankenship and Hartman 1998). Both photosystems are large multisubunit protein complexes made up of two parts, the light-harvesting antenna and the reaction center (Gao et al. 2018). The function of the antenna complex is to scaffold pigments that absorb and funnel light energy to the reaction center core (Gao et al. 2018). The reaction center uses this light energy for charge separation, which ultimately fuels the generation of ATP and NADPH, which in turn drive downstream cellular metabolic processes.

The first step of the light-dependent reactions is catalyzed by PSII, whereby water is oxidized in the thylakoid lumen and the liberated electrons are shuttled through a variety of cofactors to plastoquinone on the stromal side of the thylakoid membrane (Barber 1998). Meanwhile, PSI catalyzes a later step where reduced plastocyanin from the cytochrome b_6_f complex is oxidized in the thylakoid lumen and the liberated electron is shuttled across the thylakoid membrane to reduce ferredoxin in the stroma (Chitnis 2001). During periods of high light, over-reduction of the internal electron transport chain can result in the formation of reactive oxygen species that damage the photosynthetic apparatus (Hideg et al. 2002; Asada 2006; Pospíšil 2016). While several subunits in the photosystem are damaged by excess light (Yao et al. 2012), a specific target of this damage is the D1 protein, conferring protective effects by reducing the extent of damage on the rest of the PSII complex (Mattoo et al. 1981; Aro et al. 1993). In particular, a frequently damaged region of D1 is the ∼40 amino acid long stroma-exposed loop between transmembrane helices D and E, aptly termed the DE-loop (Greenberg et al. 1987; Aro et al. 1993; Frankel et al. 2013). As a result of continuous photodamage, D1 needs to be constantly replaced to maintain photosystem function. Accordingly, D1 has the highest turn-over rate of all photosystem proteins and one of the highest expression levels found in nature (Kettunen et al. 1997; Li et al. 2017; Forsythe et al. 2022).

Eukaryotes gained the capacity for photosynthesis ∼1.5 billion years ago when a single celled eukaryotic organism engulfed a cyanobacterium (Schwartz and Dayhoff 1978; Martin and Kowallik 1999; Hedges et al. 2004). As the cyanobacterium evolved into the semiautonomous organelle known as the plastid, its genome underwent a substantial reduction, such that it now harbors <5% of the genes found in its cyanobacterial ancestor (Blanchard and Schmidt 1995; Martin et al. 2002; Kelly 2021). Despite this large reduction, the gene content and organization has been highly conserved across the angiosperm lineage (Palmer and Thompson 1982; Jansen et al. 2007; Zhu et al. 2016; Robbins and Kelly 2023). Among the ∼80 protein-coding genes that have remained in the plastid genome many encode core photosystem reaction center proteins. These are single-copy genes, and each has been subject to a low rate of evolution experienced by the plastid genome (Wolfe et al. 1987; Smith 2015), a direct result of high copy number of the plastid genome and consequently high levels of gene conversion eliminating spontaneous mutations (Birky and Walsh 1992; Khakhlova and Bock 2006). Variations in gene expression, amino acid composition, and genome organization have further constrained the rate of molecular evolution of genes in the plastid genome (Robbins and Kelly 2023). Together, these factors have resulted in the plastid-encoded photosystem genes being some of the slowest evolving genes found in nature (Cardona et al. 2019; Oliver et al. 2021, 2023; Bouvier et al. 2024). Given the low rate of molecular evolution, it is unknown whether the photosystems have been adaptively evolving during the radiation of the angiosperms, which components of the photosystems have experienced higher or lower rates of adaptive evolution, and how this change has altered the properties of the photosystems themselves.

Here, we present a molecular evolution analysis at the single residue resolution of the plastid-encoded photosystem genes from 773 angiosperm species. We have focused on the angiosperm clade as it contains the majority of crop plants, including food plants for direct human consumption, animal feed, biofuels, textiles, building materials, and pharmaceuticals (Raskin et al. 2002; Leff et al. 2004; Mahapatra et al. 2021). Consequently, understanding the evolution of their photosynthetic apparatus holds most potential to inform future crop improvement strategies. We uncovered a widespread occurrence of hallmarks of adaptive evolution, namely positive selection and recurrent evolution, in all photosystem genes. Through analysis of the location and type of adaptive changes that occurred, we reveal how these changes are predicted to have had a significant impact on protein–protein interfaces but not on cofactor binding within the photosystems. Moreover, we reveal a concerted suite of changes that are modeled to destabilize PSII interactions with the D1 protein thereby reducing the energetic barrier for D1 turn-over and photosystem repair.

## Results

### There is substantial variation in extent of evolution between photosystem subunits

**Figure 1.**
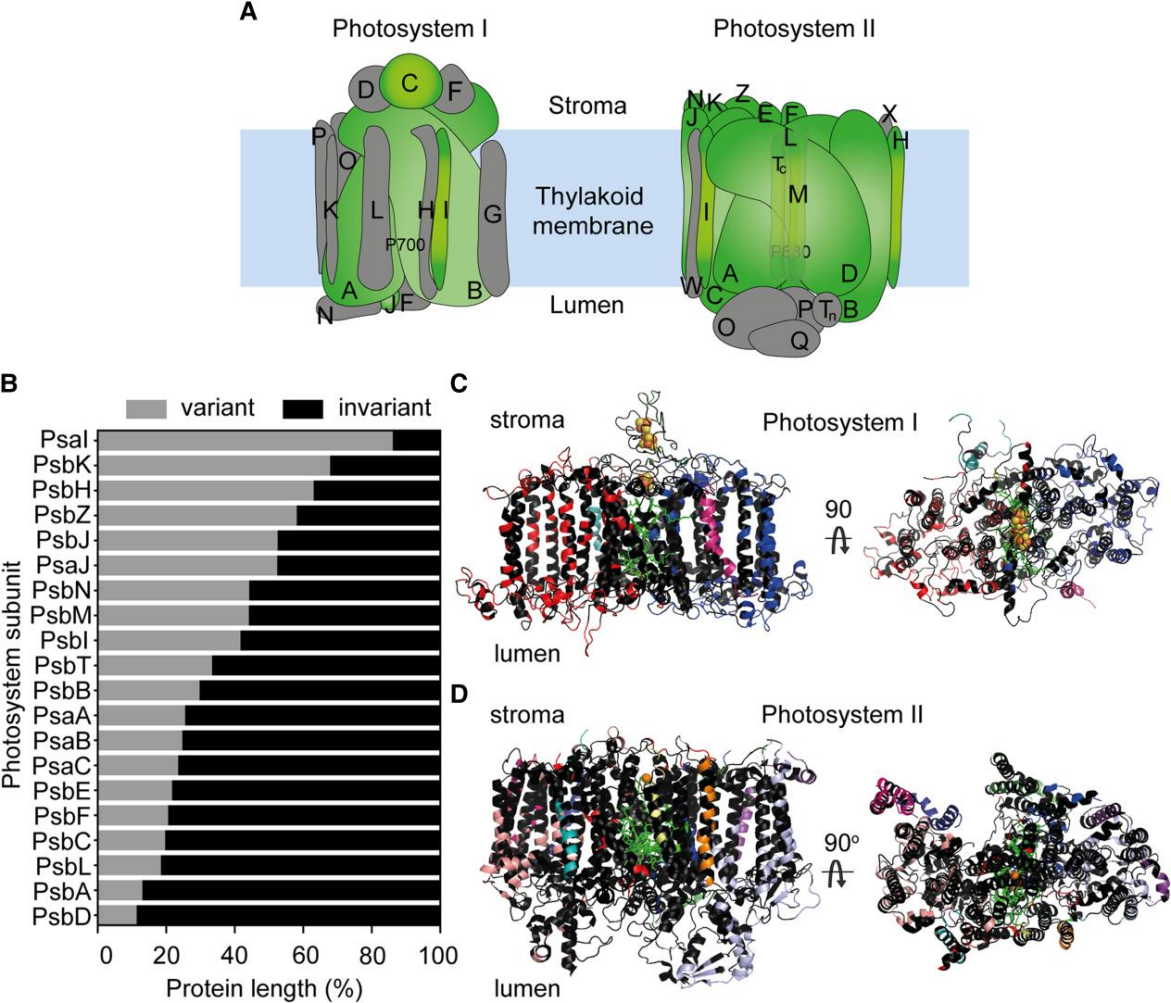
Highly conserved regions of the plastid-encoded photosystem proteins in angiosperms. **A)** Schematics of the PSI (left) and PSII (right) reaction centers with the proteins encoded by the 20 genes analyzed in this study highlighted in green. Proteins in gray were not ubiquitously conserved in the plastid genome among the species analyzed. **B)** Bar chart showing the percentage of each protein that was variant (gray) versus invariant (black) in the 773 angiosperm species dataset. Genes are ordered by decreasing percentage of protein that is variable from top to bottom. **C)** Crystal structure of the proteins analyzed in PSI with invariant residues highlighted in black. Red, PsaA; blue, PsaB; dark green, PsaC; pink, PsaI; and teal, PsaJ. For reference, the electron transfer pathway pigments are shown as green sticks and iron–sulfur centers as orange/yellow spheres. **D)** Crystal structure of the genes analyzed in PSII with invariant residues highlighted in black. Red, PsbA; light blue, PsbB; salmon, PsbC; blue, PsbD; pale green, PsbE; dark green, PsbF; purple, PsbH; teal, PsbI; dark blue, PsbK; green, PsbL; orange, PsbM; pale yellow, PsbT; and pink, PsbZ. Pigments are shown as green sticks and the Fe^2+^ ion as an orange sphere.

The chloroplast genome in angiosperms contains 20 genes encoding photosystem proteins, 5 from PSI (*psaA*, *psaB*, *psaC*, *psaI*, and *psaJ*) and 15 from PSII (*psbA*, *psbB*, *psbC*, *psbD*, *psbE*, *psbF*, *psbH*, *psbI*, *psbJ*, *psbK*, *psbL*, *psbM*, *psbN*, *psbT*, and *psbZ*) (Fig. 1A). To analyze the molecular evolution of these genes, a robust phylogeny was built from a concatenated multiple sequence alignment of all ubiquitously conserved protein-coding sequences from 773 complete angiosperm plastid genomes (see methods). For each photosystem gene, ancestral sequence reconstructions were performed for all 771 internal nodes in the phylogenetic tree, and all nonsynonymous substitutions were identified and mapped to branches in the tree.

**Figure 2.**
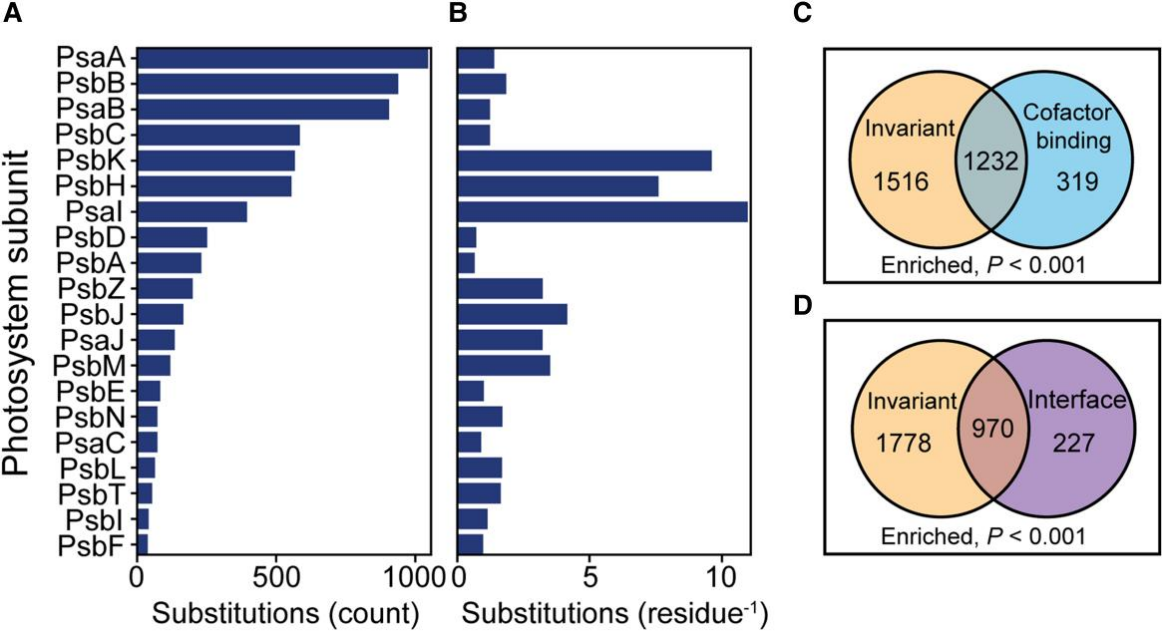
Amino acid substitutions in plastid-encoded photosystem genes during radiation of the angiosperms. **A)** Horizontal bar chart of the raw count of amino acid substitutions inferred to have occurred across the phylogeny for each protein. Proteins are ordered by decreasing number of amino acid substitutions from top to bottom, respectively. **B)** Horizontal bar chart of the substitution count per residue for each protein. Proteins are ordered as in **(A)**. **C)** Venn diagram showing the overlap between invariant residues (yellow) with cofactor binding residues (blue). **D)** Venn diagram showing the overlap between residues that are invariant (yellow) with those that are at the protein–protein interfaces between subunits (purple). *P*-values shown below Venn diagrams are the results of hypergeometric tests.

Although 74% (2,850/3,870) of all residues exhibited no variation (*d*_N_ = 0, Fig. 1B–D), 6,732 amino acid substitutions were identified at the remaining 1,020 residues, with substitutions occurring in all 20 photosystem proteins (Fig. 2A). Moreover, there was a 17-fold difference between the photosystem protein with the largest and fewest amino acid substitutions per residue across the 773 species phylogeny, corresponding to 11 substitutions per residue for PsaI (encoded by *psaI*) and 0.65 substitutions per residue for D1 (*psbA*), respectively (Fig. 2B). Thus, although the plastid-encoded photosystem genes have evolved slowly during angiosperm evolution, evolution has occurred and there is significant variation in evolutionary rate between photosystem components.

### Despite strong functional constraint there is evidence of widespread adaptive evolution in the photosystems of angiosperms

To gain insight into the functional constraints and adaptive evolution of the photosystems in angiosperms, we first analyzed the sites that exhibited no variation in the 773 species dataset. This revealed that there was an enrichment of invariant sites in the cofactor binding residues of the photosystems (hypergeometric test, *P* < 0.001; Fig. 2C). Furthermore, there was a significant enrichment of invariant sites at subunit interfaces in both photosystems (hypergeometric test, *P* < 0.001; Fig. 2D). Thus, the requirement to bind cofactors and form intersubunit interfaces has resulted in severe constraint on the extent of molecular evolution of the photosystem genes and can explain 58% of all invariant sites in these complexes.

**Figure 3.**
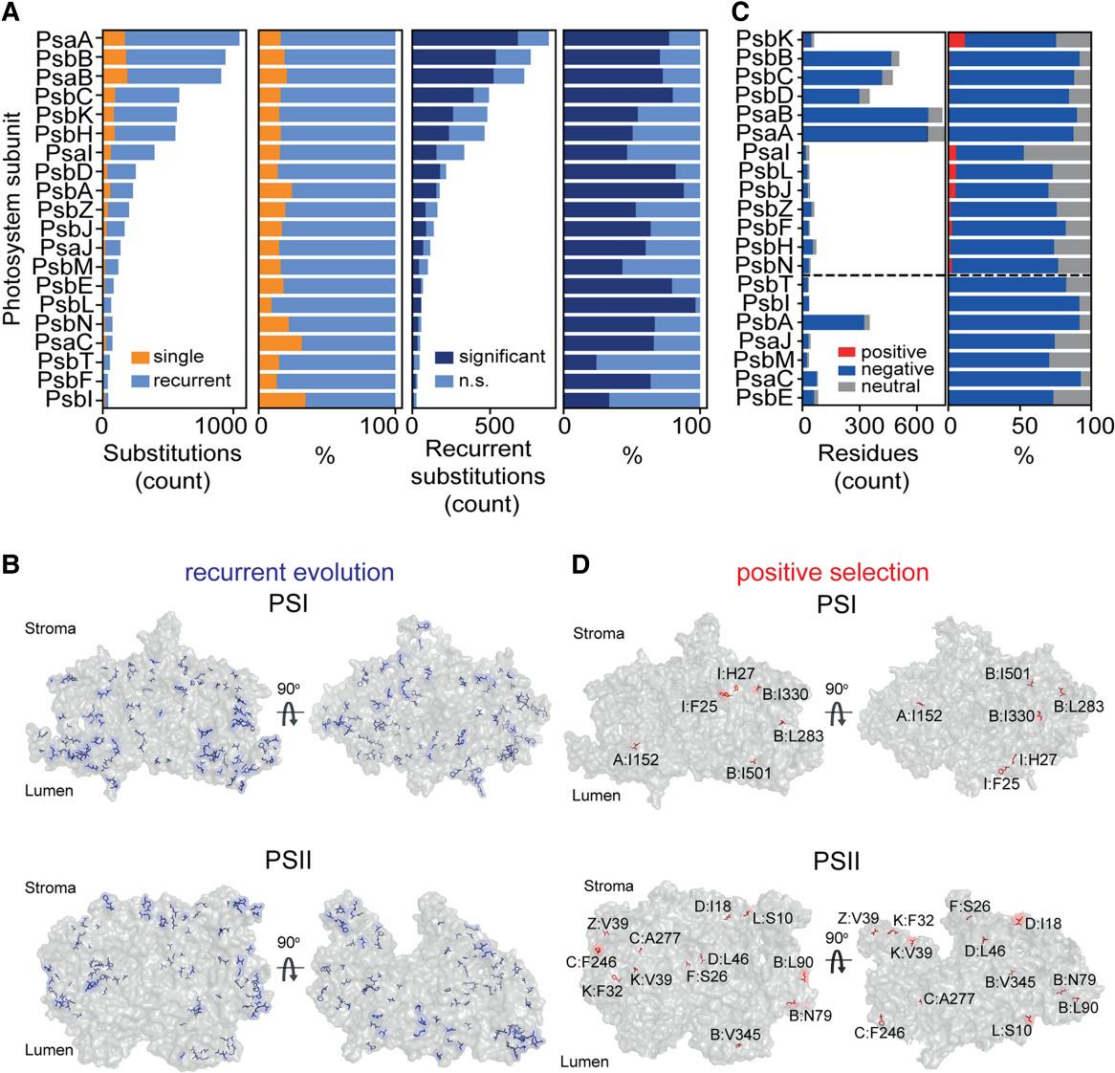
Adaptive evolution in the plastid-encoded photosystem proteins during the radiation of the angiosperms. **A)** Left two bar charts show amino acid substitution counts (left) and percentage of substitutions (right) that are single occurrence (orange) or recurrent (blue), i.e. a specific amino acid replacement that has occurred once or more than once at a given residue, respectively. Proteins are ordered by decreasing number of substitutions inferred to have occurred. Right two bar charts show the proportion of recurrent substitutions that occurred more frequently than expected by chance (*P* < 0.001, Monte Carlo test, dark blue) as compared to those that did not (light blue). Raw counts are shown on the left and percentage of recurrent substitutions shown on the right. **B)** Bar charts showing the number (left) and percentage (right) of residues under positive selection (red), purifying selection (blue), or subject to neither (gray). Proteins are ordered by decreasing number of sites under positive selection from top to bottom. All proteins below the dashed line have no sites under positive selection. **C** and **D)** Structures of PSI and PSII with modeled sites of recurrent evolution highlighted in blue and sites subject to positive selection shown in red, respectively.

Although sites involved in cofactor binding and subunit interfaces exhibited strong functional constraint, substantial variation is present. This raised the question as to whether this variation exhibits evidence of adaptive evolution, and if so, whether this change has altered the properties of the photosystems. As recurrent evolution and positive selection are widely regarded as signatures of adaptation to common selective constraints (Yang et al. 2000; Wood et al. 2005; Kapralov and Filatov 2007; Losos 2011; Stern 2013; Ord and Summers 2015), we sought to determine whether either of these hallmarks could be detected at variable sites in photosystem genes. Given that we had precisely mapped all amino acid substitutions to specific branches in the angiosperm phylogeny, it was possible to directly address the question as to whether any of the amino acid substitutions were recurrent in multiple branches of the phylogeny (a recurrent amino acid substitution is here defined as an amino acid substitution X → Y, where X ≠ Y that has occurred more than once at a given site in a protein sequence at a frequency greater than expected by chance; see methods). Remarkably, 54% (3,620/6,522) of all amino acid substitutions were recurrent and unlikely to have evolved by stochastic co-occurrence of random mutations in the absence of selection (*P* < 0.001, Fig. 3A). These recurrent substitutions were located at 275 sites across all 20 proteins and comprised 386 unique patterns of amino acid substitution (Fig. 3B, Supplementary Table S1). Similarly, there was strong evidence for pervasive positive selection acting at 35 sites in 13 genes (Fig. 3C and D, Supplementary Table S2). Moreover, 30 of these 35 positively selected sites were also identified in the set of recurrent substitutions demonstrating a substantial overlap between these two hallmarks of adaptive evolution (hypergeometric test, *P* < 0.001; Supplementary Fig. S1). Consistent with this, there is a significant depletion of recurrent substitutions at sites that are detected as evolving under purifying selection (hypergeometric test, *P* < 0.001; Supplementary Fig. S1). Thus, there is evidence that 280 sites across all 20 plastid encoded photosystem genes may have evolved adaptively during the radiation of the angiosperms.

### There is no significant difference in the extent of adaptation between photosystem complexes during angiosperm evolution

**Figure 4.**
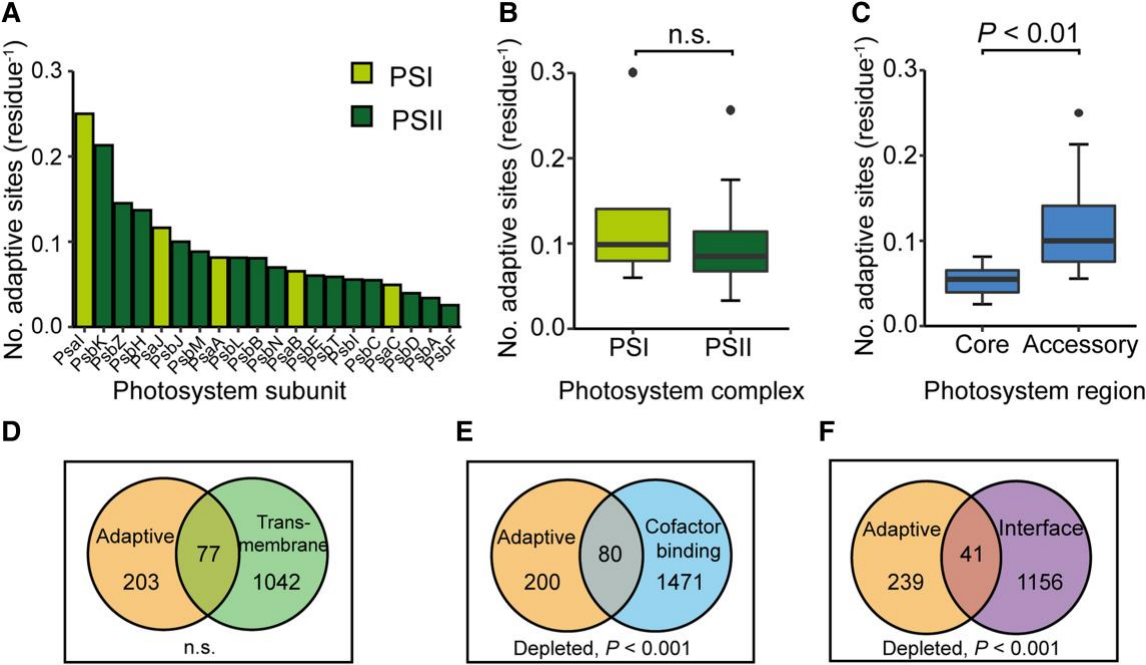
Analyzing regional and structural enrichment of adaptive evolution in the photosystems. **A)** Bar chart showing the number of sites under adaptive evolution in the 20 photosystem proteins analyzed corrected for protein length in decreasing order from left to right. Proteins in PSI and PSII are in light green and dark green, respectively. **B)** Boxplot showing data in **(A)** grouped by photosystem complex. Two-sided two-sample *t*-test, *P* > 0.05 (PSI *n* = 5 and PSII *n* = 15). **C)** Boxplot showing data in **(A)** grouped by photosystem region (Core *n* = 9 and Accessory *n* = 11). Boxplots show median (dark line), shaded areas encompass the second and third quartiles, the whiskers indicate the boundaries of the first and fourth quartiles, and dots indicate outliers. Core proteins included PsaA, PsaB, PsaC, PsbA, PsbD, PsbC, PsbD, PsbE, and PsbF. Peripheral proteins included PsaI, PsaJ, PsbH, PsbI, PsbJ, PsbK, PsbL, PsbM, PsbN, PsbT, and PsbZ. *P*-value shown derived from two-sided two-sample *t*-test. **D–F)** Venn diagrams showing the overlap between adaptively evolving sites (orange) and transmembrane residues (green), cofactor binding residues (blue) and intersubunit residues (purple), respectively. *P*-values shown are the results of hypergeometric tests.

We next sought to determine whether adaptive evolution had been more prevalent in either the proteins of PSI or PSII during the evolution of the angiosperms. Among the 280 adaptively evolving sites, 127 were in PSI and 153 were in PSII. Given the number of adaptively evolving sites in a protein was strongly correlated to its length (*R*^2^ = 0.67, *P* < 0.001, Supplementary Fig. S2), we compared the number of adaptively evolving sites per residue between subunits of PSI versus PSII. Although this uncovered a 10-fold difference between the proteins with the highest and lowest levels of adaptive evolution corresponding to 0.250 adaptive sites per residue in PsaI and 0.026 adaptive sites per residue in PsbF, respectively (Fig. 4A), there was no significant difference in the level of adaptive evolution between PSI and PSII subunits (*t*-test, *P* < 0.05; Fig. 4B). Thus, the levels of adaptive evolution experienced by PSI and PSII have been equivalent during the radiation of the angiosperms.

### Adaptive evolution has been limited in regions of the photosystems required for energy and electron transfer

Given that the extent of adaptive evolution was comparable between photosystem complexes, we next sought to investigate whether there was regional enrichment of adaptation within the photosystem structures. Specifically, we wanted to explore whether more adaptation had occurred in the core versus accessory proteins of the photosystem reaction centers, transmembrane versus extramembrane regions or functional regions such as cofactor binding or protein–protein interfaces. To test the former, core proteins were defined as subunits binding the redox cofactors forming the electron transport chain (PsaA, PsaB, PsaC, D1, and D2), internal antenna proteins (PsbB and PsbC), and cytochrome b559 (PsbE and PsbF). Meanwhile, accessory proteins included the remaining 11 low molecular weight (<10 kDa) proteins that are on the periphery of the core reaction center (PsaI, PsaJ, PsbH, PsbI, PsbJ, PsbK, PsbL, PsbM, PsbN, PsbT, and PsbZ). This revealed adaptive sites were significantly more prevalent in the accessory proteins compared to the core proteins of the photosystem reaction centers, with a mean fold difference of 2.2 (*t*-test, *P* < 0.01; Fig. 4C). Meanwhile, there was no significant enrichment of adaptive sites in either the transmembrane or extramembrane regions of the photosystem proteins (Fig. 4D). Lastly, adaptive sites were significantly depleted in both cofactor binding and intersubunit interface residues (hypergeometric test, *P* < 0.001; Fig. 4E and F), making up 29% and 15% of adaptive sites, respectively. Thus, adaptive evolution has been constrained in regions of high functional constraint including the subunits responsible for energy and electron transfer and residues coordinating cofactors and protein–protein interfaces.

### Adaptive evolution has weakened D1 protein interfaces during angiosperm evolution

**Figure 5.**
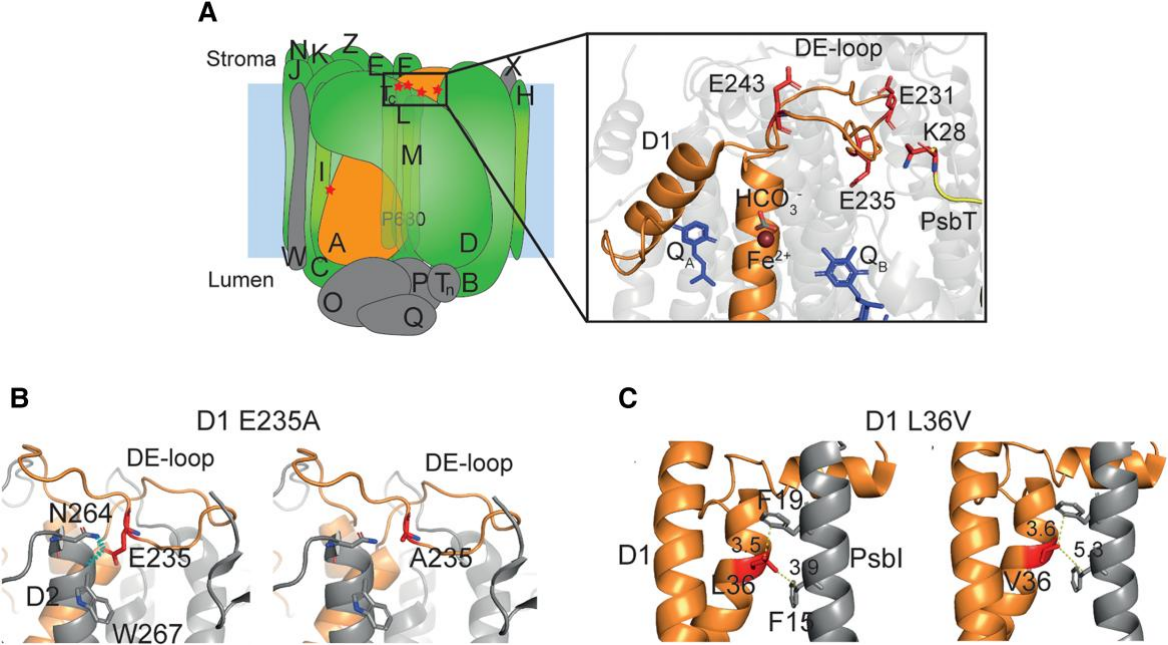
The position and impact of adaptive substitutions on intersubunit interactions with the D1 (*psbA*) protein. **A)** Schematic mapping the location of substitutions that destabilize intersubunit interactions with D1. The D1 protein is shown in orange for clarity and sites of substitutions are marked with a red star. A close-up view of the cluster of destabilizing substitutions in/around the DE-loop of D1 in the 7OUI PSII structure is also provided. Residues at sites of destabilizing substitutions are shown as red sticks. For reference, the two plastoquinones (Q_A_ and Q_B_), Fe^2+^, and HCO_3_^−^ involved in electron transfer are also shown. **B)** Loss of hydrogen bonds (dashed cyan lines) upon E → A substitution between D1 235 and D2 (*psbD*) N264 and W267. **C)** Reduced hydrophobic interactions upon L → V substitution at 36 in D1 with F15 and F19 in PsbI. Yellow dashed lines show distance between residues given in Å.

Despite being depleted at cofactor binding and intersubunit interface residues, we next sought to assess the impact of the adaptive substitutions that had occurred within these respective regions. To do this, we calculated the change in the interaction energy at the protein-cofactor and protein–protein interfaces, respectively, by analyzing alterations in structural features and empirical energy terms at the interface upon substitution. We found none of the 80 adaptive substitutions at cofactor binding sites were predicted to significantly affect ligand binding (Supplementary Table S1). However, of the 41 sites at intersubunit interfaces 10 substitutions were predicted to significantly stabilize their respective intersubunit interface and 9 substitutions were predicted to significantly destabilize their respective intersubunit interface (Table 1 and Supplementary Table S1). Of particular note was that all recurrent substitutions at protein–protein interfaces involving the D1 protein (*psbA*) were predicted to be destabilizing (one-sample *t*-test, *P* < 0.05; Supplementary Fig. S3 and Table S1), weakening the association of this subunit with the remainder of the photosystem complex. Mapping these sites onto the PSII structure revealed that they were primarily located either in, or in contact with, the stromal DE-loop of D1 (*psbA*), a region of known importance for binding and positioning the bicarbonate ion and nonheme iron (Fig. 5A). These adaptive changes weakened the interface of the core complex with D1 through a variety of mechanisms. For example, in the DE-loop the recurrent substitution E235A in D1 (*psbA*) results in the loss of polar contacts between E235 in D1 with N264 and W267 in the D2 (*psbD*) protein (Fig. 5B). Similarly, although it is not located in the DE-loop region, the L36V substitution in D1 (*psbA*) reduces hydrophobic contacts with F15 and F19 in the transmembrane helix of PsbI (*psbI*) (Fig. 5C). Together, these results indicate that the photosystems in angiosperms have been adaptively evolving to make it easier to dissociate D1 from the core photosystem complex. This finding is consistent with D1 being the primary recipient of photodamage in PSII (Mattoo et al. 1981; Aro et al. 1993), having the highest turn-over rate of all subunits in PSII (Kettunen et al. 1997; Li et al. 2017; Forsythe et al. 2022), and thus a requirement for continual removal and replacement of D1 to restore photosystem function.

**Table 1.** Adaptive substitutions that substantially affect protein–protein interface stability (|ΔΔG_PPI_| ≥ 0.5 kcal mol^−1^).

Protein	Substitution	Protein–protein interface	DDGPPI (kcal mol^−1^)
PsaB	M640T	**B**:A	−6.15
PsbF	S26F	**F**:E	−2.7
PsaI	S25F	**I**:L	−1.67
PsbE	D68N	D:**E**:U	−1.53
PsaC	H71P	**C**:B	−1
PsbE	S72P	**E**:D	−0.66
PsbL	N10S	B:**L**:M	−0.57
PsaJ	I4L	**J**:F	−0.54
PsaB	N636T	**B**:A	−0.53
PsbB	C124T	**B**:H	−0.53
PsbL	S10N	B:**L**:M	0.57
PsbD	I18T	**D**:X	0.66
PsbE	P72S	**E**:D	0.66
PsbA	L36V	**A**:I	0.95
PsbT	K28T	A:**T**:D	1.36
PsbA	E235A	**A**:D	1.43
PsbA	E231Q	**A**:B	2.43
PsbB	A471S	**B**:D	4
PsaC	H71N	**C**:B	4.23

Bold letters indicate the subunit containing the substitution.

## Discussion

Oxygenic photosynthesis underpins nearly all life on earth. At the heart of the process are the photosystems, the molecular machines that facilitate the first step of photochemical conversion of energy from the sun into chemical energy that powers cellular metabolic processes. Here, we investigate the molecular evolution the plastid-encoded photosystem genes in angiosperms. We show that hallmarks of adaptive evolution, namely positive selection and recurrent evolution, are widespread in the photosystem subunits of angiosperms. Moreover, we reveal a concerted suite of changes are predicted to have destabilized the interaction of PSII with its D1 subunit reducing the energetic barrier for D1 turn-over and photosystem repair.

The finding that all adaptive substitutions at the intersubunit interfaces with D1 (*psbA*) were predicted to destabilize the protein–protein interactions is consistent with extremely high turnover of the subunit (Kettunen et al. 1997; Li et al. 2017; Forsythe et al. 2022), and the concomitant requirement to constantly remove and replace damaged D1 protein. Four of the five of these substitutions were found to be clustered in or around the stroma-exposed DE-loop in D1 (D1: E231Q, E235A, E243G and PsbT: K28T), which has been attributed two main functions. The first is that it provides two ligands (E244 and Y246) that indirectly coordinate the nonheme iron positioned between Q_A_ and Q_B_ via positioning a bicarbonate ion (Gao et al. 2018). Second, the region between residues 238 and 248 in the DE-loop is a site of cleavage during D1 degradation, yielding the 23 kDa fragment (Greenberg et al. 1987). Moreover, it has been postulated that a damage induced increase in mobility of the DE-loop may signal PSII repair (Forsman and Eaton-Rye 2021). In this context, it is interesting to note that three of the adaptive substitutions found to destabilize a D1 intersubunit interface were located in the DE-loop region of D1, E231Q, E235A, and E243G. Interestingly, it was shown that mutagenesis at two of these sites (E231D and E243Q) did not affect electron transfer but did substantially reduce the half-life of D1 (Mulo et al. 1997). Given that electron transfer was not affected, the bicarbonate ion and nonheme iron must have still been successfully bound to the photosystem, suggesting the DE-loop has maintained a mostly correct conformation. If this is the case, the reduced half-life cannot be the result of an aberrant DE-loop conformation signaling immediate PSII degradation. An alternative hypothesis is that these substitutions result in less photodamage being required to initiate removal and replacement of D1, due to a reduction in the energy required to remove D1 from PSII. It is most parsimonious to assume that the adaptive substitutions we uncovered here act in a similar manner.

We also identified a further adaptive substitution, PsbT K28T, which is located adjacent to the DE-loop of D1 and predicted in this analysis to weaken the D1:PsbT protein–protein interface. Importantly, this adaptive substitution should not disrupt important hydrophobic interactions that occur between the neighboring residues in PsbT (P27 and I29) with F239 in the DE-loop of D1. Without these hydrophobic interactions, the DE-loop has increased flexibility that leads to bicarbonate dissociation and slowed electron transfer between Q_A_^−^ and Q_B_ (Forsman and Eaton-Rye 2021). This increased flexibility and resultant aberrant conformation of the DE-loop is thought to be the cause of an increase in turnover of D1 (Forsman and Eaton-Rye 2021). Thus, taken together the findings presented here indicate that the K28T substitution in PsbT is adaptive as it weakens the connection between PsbT and D1 without disrupting the DE-loop conformation, thereby enhancing D1 repair signaling in response to photodamage.

The question arises as to why reducing the energetic barrier for D1 turn-over and PSII repair would lead to an adaptive advantage. At any given time, the proportion of damaged photosystems is a function of the incident light, the activity of photoprotective mechanisms, and the rate of photosystem repair (Su et al. 2023). Each of these factors interact to alter the proportion of PSII containing a nonfunctional D1 subunit and are thus in a nonproductive photoinhibited state. Evolution by natural selection can act to modulate each of these factors to maximize the conversion of incident light energy into chemical energy and growth. As turn-over is triggered by the detection of nonfunctional D1, reducing the energetic barrier for D1 removal and replacement would in turn increase the rate at which damaged PSII complexes can be repaired. Furthermore, as photoinhibition only occurs when the rate of repair is unable to keep up with the rate of damage (Prasil 1992), the overall effect of reducing the energetic barrier to repair would be to have a lower proportion of nonfunctional PSII complexes, thereby increasing the conversion of incident light energy into chemical energy and growth.

The recurrent substitutions identified in this study are not uniformly distributed across the phylogeny. For example, E235A in D1 was identified in species from 15 different families (Cornales, Arecales, Laurales, Chloranthales, Nymphaeales, Gentianales, Rosales, Poales, Ericales, Malvales, Caryophyllales, Zingiberales, Myrtales, Huerteales, and Vitales) while E243G in D1 was only observed in four families (Brassicales, Apiales, Rosales, and Ericales). These differences in distribution may allow the effect of these substitutions to be experimentally distinguished. In this context, it will be interesting to perform a large-scale analysis of photoinhibition on species within these groups to understand how these changes have altered the function of PSII. Specifically, it will be interesting to determine if species harboring these changes are less subject to photoinhibition across a range of different light intensities. Moreover, it will also be interesting to determine if introduction of these changes into species in which they have not evolved can result in a reduction in photoinhibition.

In this analysis, we identified that 7% of sites in the plastid-encoded photosystem proteins of angiosperms show hallmarks of adaptive evolution. Furthermore, we showed that many of these sites are located at regions of known functionality with 29% of adaptively evolving sites involved in cofactor binding and 15% of sites at intersubunit interfaces. However, while we provide biological explanations for substitutions at several adaptively evolving sites, the majority remain unexplained. For some of these unexplained sites, *in silico* prediction of the effect of substitution is impossible due to their location in regions that are not modeled in the photosystem structures. Meanwhile, for the currently unexplained sites that are resolved in the photosystem structures their beneficial trait is not obvious from analyzing the change to protein structure. Nonetheless, this study identifies several regions of the photosystems with hallmarks of adaptive evolution and further experimental investigation is required to elucidate the biological importance of these changes.

The analysis presented here focused on angiosperms as information gained here holds most potential to inform future crop improvement strategies. However, the analysis presented here will provide a foundation and point of comparison for investigation of adaptive evolution in photosystems of other lineages, such as algae or cyanobacteria, in future studies. In this context, it is interesting to note that there are differences in the repertoire of genes that constitute the photosystems in these lineages. For example, the D1 gene is encoded by a multigene family in cyanobacteria (Mulo et al. 2009). Here, different family members are differentially expressed according to environmental cues to adapt photochemical fitness to solar intensity (Vinyard et al. 2013). Given the sub-functionalization that has occurred, it may be that different gene family members are adapting along different evolutionary trajectories. For example, the high light adapted isoform may exhibit an analogous trajectory as described here for angiosperms, reducing the energetic barrier for D1 repair, while the low light adapted version may exhibit a different trend. Furthermore, comparison between recurrent substitutions in different Archaeplastida lineages may provide further insight into global trends not detectable through analysis of angiosperms alone.

Computational predictions of structural stability have been used repeatedly to inform the rational design and improvement of many proteins, for example (Yang et al. 2007; Floor et al. 2014; Goldenzweig et al. 2016; Shirke et al. 2016). However, these methods are not yet perfect (Marabotti et al. 2021) and future re-evaluation of the data presented here with more sophisticated methods may uncover additional changes to the photosystems in angiosperms not reported here.

Our findings show that all plastid-encoded photosystem proteins exhibit hallmarks of adaptive evolution during the radiation of the angiosperms. By uncovering these past trajectories, and identifying key sites that have been altered by natural selection, these findings provide substantial new insight into the present and future evolution of the photosystems in angiosperms.

## Materials and methods

### Phylogenetic tree inference

The plastid genome dataset and phylogenetic tree used in this study was the same as in (Robbins and Kelly 2023). In brief, the full set of sequenced plastid genomes was downloaded from the National Center of Biotechnology and Information (NCBI) in July 2021. Genomes were filtered so that those that did not contain the full complement of canonical plastid genes were removed, resulting in a dataset containing nucleotide, and corresponding protein sequences, for 69 plastid genes for 773 angiosperm species. For each of the 69 genes, a protein multiple sequence alignment was generated using MAFFT L-INS-I (Katoh and Standley 2013) and was used to guide an accurate nucleotide alignment using Pal2Nal (Suyama et al. 2006). The nucleotide alignments were then trimmed to remove columns containing >90% gaps and concatenated together and used to identify the best-fit model of nucleotide evolution using IQ-TREE's inbuilt ModelFinder (Kalyaanamoorthy et al. 2017). The best-fitting model of nucleotide evolution identified was GTR+F+R7. IQ-TREEs ultrafast bootstrapping method with 1,000 replicates was then used to infer a maximum likelihood phylogenetic tree using the concatenated nucleotide sequence and GTR+F+R7 model of evolution (Hoang et al. 2018). The tree was than manually rooted on the Nymphaeales clade, as they are a sister lineage to the rest of the sampled angiosperms in the dataset. The species tree and alignment files are available to download from Zenodo at https://doi.org/10.5281/zenodo.13750849

### Inferring nonsynonymous substitutions and calculating the rate of nonsynonymous substitution

Among the 69 ubiquitously conserved genes above were 20 genes that encode proteins that participate in the photosystem reaction centers, 5 for PSI (*psaA*, *psaB*, *psaC*, *psaI*, and *psaJ*) and 15 for PSII (*psbA*, *psbB*, *psbC*, *psbD*, *psbE*, *psbF*, *psbH*, *psbI*, *psbJ*, *psbK*, *psbL*, *psbM*, *psbN*, *psbT*, and *psbZ*). Ancestral state reconstructions were generated for each of the 20 photosystem genes for every node in the maximum likelihood phylogenetic tree using the GTR+F+R7 model of evolution in IQ-TREE (Nguyen et al. 2014). Nonsynonymous substitutions at every internal branch in the tree were inferred by assessing all child-parent relationships in the phylogeny, excluding the Nymphaeles outgroup, using a bespoke Python script (https://github.com/ElizabethRobbins/Identify_amino_acids_substitutions_in_phylogeny.git). These were translated into amino acid replacements at every residue in the protein alignment. Substitutions were tabulated in matrices where columns represent the parent residue identity and rows represent the child residue. These matrices are summarized in Supplementary Table S3. The gene trees, multiple sequence alignments, and ancestral state sequences are available to download from Zenodo at https://doi.org/10.5281/zenodo.13750849. Site-wise rates of nonsynonymous mutation (*d*_N_) were determined using the FEL method from HYPHY's software suite (Kosakovsky Pond and Frost 2005).

### Identifying cofactor binding and protein–protein interface residues

Residues involved in cofactor binding and protein–protein interactions were identified using the 5L8R PSI from *Pisum sativum* (2.6Å resolution) (Mazor et al. 2017) and 7OUI PSII from *Arabidopsis thaliana* (2.79Å resolution) (Graça et al. 2021) structures. To identify cofactor binding residues, all pigments and cofactors associated with the 20 subunits analyzed were identified in the photosystem structures. For PSI, this included 84 chlorophyll a molecules, 2 phylloquinones, 16 beta-carotenes, 1 (3R,3′R,6S)-4,5-didehydro-5,6-dihydro-beta,beta-carotene-3,3′-diol, 4 1,2-dipalmitoyl-phosphatidyl-glycerole, 5 1,2-distearoyl-monogalactosyl-diglyceride, 3 digalactosyl diacylglycerol, L, 1 Cl^−^, 1 Ca^2+^, and 3 iron–sulfur centers. Meanwhile, for PSII this included 35 chlorophyll a molecules, 4 chlorophyll b molecules, 2 Ca^2+^, 1 Cl^−^, 11 beta-carotene, 1 HCO_3_^−^, 4 digalactosyl diacylglycerol, 6 1,2-distearoyl-monogalactosyl-diglycerise, 2 pheophytin, 2 plastoquinone, and 4 sulfaquinovosyldiacylglycerol molecules. Residues coordinating these pigments and cofactors were identified by conducting a search for residues with at least one heavy atom <4Å from an atom in a pigment or cofactor using the NeighbourSearch function in the Bio.PDB package (Hamelryck and Manderick 2003) (Supplementary Fig. S4 and Table S4). Protein–protein interface residues were identified using the EMPL-EBI PDBsum database (Supplementary Fig. S5 and Table S5) (Laskowski et al. 2018).

### Recurrent evolution and positive selection

Recurrent substitutions were defined as amino acid substitutions, i.e. X −> Y, where X ≠ Y, that were inferred to have occurred more than once at a specific residue in the phylogeny. Given that recurrent substitutions could have arisen from chance, a Monte Carlo approach was developed to assess whether they were likely to occur by chance given the inferred model of sequence evolution and the phylogenetic relationships between species encapsulated by the tree. To do this, the best-fitting model of protein sequence evolution was identified from the concatenated alignment of all 69 conserved plastid protein sequences and the species tree using IQ-TREE's inbuilt ModelFinder (Kalyaanamoorthy et al. 2017). The best-fitting model identified was JTT+F+R7. Branch lengths for protein trees that were constrained to the topology of the species tree were then inferred for each of the 20 photosystem protein alignments using the JTT+F+R7 model of protein evolution. Protein sequences for extant species were then simulated for each of the 20 photosystem genes from the protein tree and ancestral node sequence using IQ-TREE's alignment simulator, with 1,000 replicates. For each of the simulated alignments, ancestral sequences were inferred, and amino acid substitutions counted as described above. Only the recurrent substitutions that had not occurred at the same or higher frequency in any of the 1,000 simulations of protein evolution was deemed significant and a hallmark of adaptive evolution.

Sites under positive selection were identified using the FUBAR method from the HYPHY software package (Murrell et al. 2013). Sites with a posterior probability that the rate of nonsynonymous substitution is greater than the rate of synonymous substitution (β > α) of >0.9 were defined as under positive selection. Conversely, sites under purifying selection were identified as having a posterior probability that α > β >0.9.

### Modeling the effect of amino acid substitutions on cofactor binding and protein–protein interfaces

Single amino acid substitutions were introduced into the PSI (5L8R) and PSII (7OUI) structures using PyMol. To estimate the effect of a substitution on cofactor binding, the change in Gibb's free energy of binding (Δ*G*_binding_) between protein and ligand was calculated for the wild-type and mutated structures using the PRODIGY-LIG webserver (Vangone et al. 2018). From these, the change in Δ*G*_binding_ between the mutated and wild-type structure (ΔΔ*G*_binding_) can be calculated, whereby a negative value indicates an increased binding affinity, and vice versa. Similarly, to estimate the effect of substitution at a protein–protein interface, the change in Gibb's free energy of interaction (Δ*G*_PPI_) was calculated using BAlaS for both the wild-type and mutated protein structures (Wood et al. 2020). As before, the change in Δ*G*_PPI_ upon mutation (ΔΔ*G*_PPI_) was then calculated. For both ΔΔ*G*_binding_ and ΔΔ*G*_PPI_ only values < −0.5 or >0.5 kcal mol^−1^ were considered to substantially alter interface interactions (Benevenuta et al. 2022).

### Accession numbers

Sequence data from this article can be found in the NCBI under accession numbers provided in Supplementary File 1.

## Supplementary Material

koae281_Supplementary_Data

## Data Availability

All data are provided in the Supplementary material or is available from Zenodo at https://doi.org/10.5281/zenodo.13750849

## Supplementary data

The following materials are available in the online version of this article.

**Supplementary File 1.** Accession numbers for all sequences used in this analysis.

**Supplementary Figure S1.** Venn diagrams showing the overlap between sites with recurrent substitutions and positive selection, and purifying selection.

**Supplementary Figure S2.** The relationship between the number of inferred nonsynonymous substitutions and protein length.

**Supplementary Figure S3.** Plot showing the change in intersubunit interaction energy upon adaptive substitutions at a PsbA (D1) interface.

**Supplementary Figure S4.** Protein structures showing the cofactor interacting residues in the plastid-encoded photosystem proteins.

**Supplementary Figure S5.** Protein structures showing intersubunit interface residues in the plastid-encoded photosystem proteins.

**Supplementary Table S1.** All significant recurrent substitutions inferred to have occurred in the phylogeny for all 20 photosystem proteins.

**Supplementary Table S2.** Positive selection sites identified in the 20 photosystem genes.

**Supplementary Table S3.** All substitutions inferred to have occurred in the phylogeny for all 20 photosystem proteins.

**Supplementary Table S4.** List of intersubunit interface residues identified in the photosystem proteins analyzed.

**Supplementary Table S5.** List of cofactor binding residues identified in the photosystem proteins analyzed.

## Dive Curated Terms

The following phenotypic, genotypic, and functional terms are of significance to the work described in this paper:

proteins CHEBI: CHEBI:36080

ATP CHEBI: CHEBI:15422

NADPH CHEBI: CHEBI:16474

PsbZ Gramene: ATCT00300
PsbZ Araport: ATCT00300
