# Supplementary material for: Widespread adaptive evolution in angiosperm photosystems provides insight into the evolution of photosystem II repair
Source: Plant Cell. 2024 Oct 15;37(1):koae281. doi: 10.1093/plcell/koae281 (PMC11663578; doi:10.1093/plcell/koae281)
Supplement: koae281_Supplementary_Data [file koae281_supplementary_data.zip › Supplementary Figures.pdf]

Supplementary Figures

Supplementary Figure S1

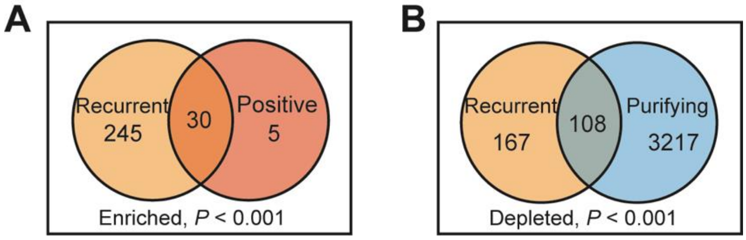

Supplementary Figure S1. Venn diagrams showing the overlap between sites with recurrent substitutions (orange) and positive selection (red) (**A**), and purifying selection (blue) (**B**). *P*-values shown are the results of hypergeometric tests.

Supplementary Figures

Supplementary Figure S2

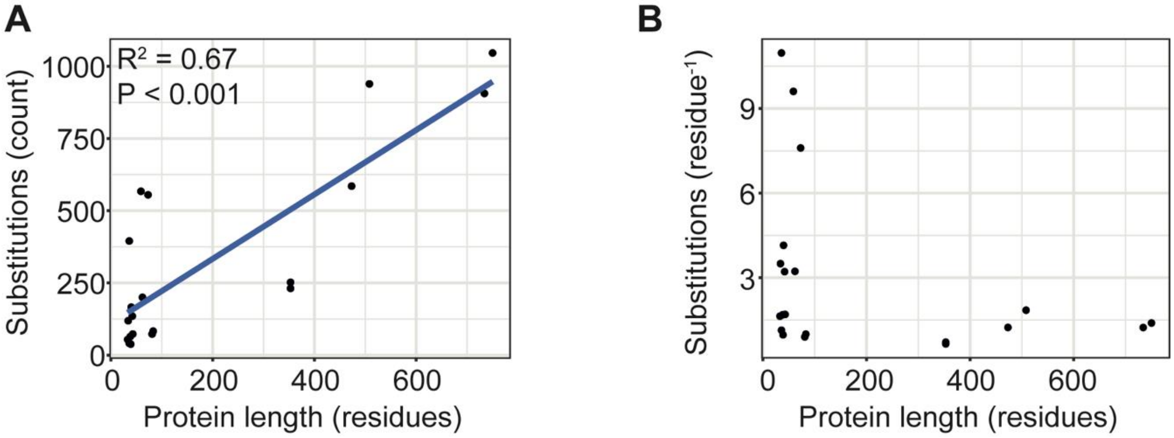

Supplementary Figure S2. The relationship between the number of inferred non-synonymous substitutions and protein length. **A**) Scatter plot of the non-synonymous substitution count per protein with the length of the protein in residues. A linear regression line is shown in blue with the associated R^2 and *P* values given. **B**) Scatter plot of the non-synonymous substitutions per residue versus protein length.

Supplementary Figures

Supplementary Figure S3

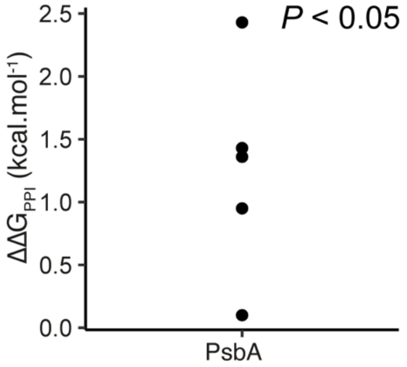

Supplementary Figure S3. Plot showing the change in inter-subunit interaction energy resulting from adaptive substitutions at a PsbA (D1) interface (PsbA L36V, E231Q, E235A, E243G; PsbT K28T). *P*-value indicates the result of a two-sided one-sample t-test.

Supplementary Figures

Supplementary Figure S4

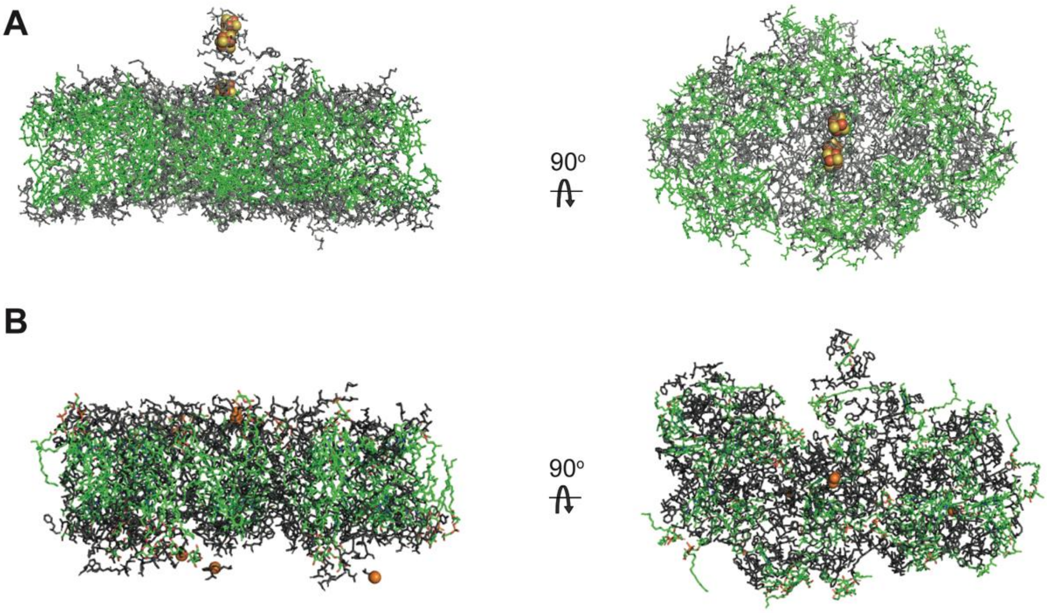

Supplementary Figure S4. Protein structures showing the cofactor interacting residues in the plastid-encoded photosystem proteins. **A**) Analysis of the 5L8R structure of photosystem I. Cofactors associated with PsaA, PsaB, PsaC, PsaI or PsaJ are shown in green. The 832 residues in these proteins that have an atom within 4Å of a cofactor atom are shown in grey. **B**) Analysis of the 7OUI structure of photosystem II. Cofactors associated with PsbA, PsbB, PsbC, PsbD, PsbE, PsbF, PsbH, PsbI, PsbK, PsbL, PsbM, PsbT or PsbZ are shown in green or as orange spheres for small molecules and atoms. The 719 residues in these proteins that have an atom within 4Å of a cofactor atom are shown in grey.

Supplementary Figures

Supplementary Figure S5

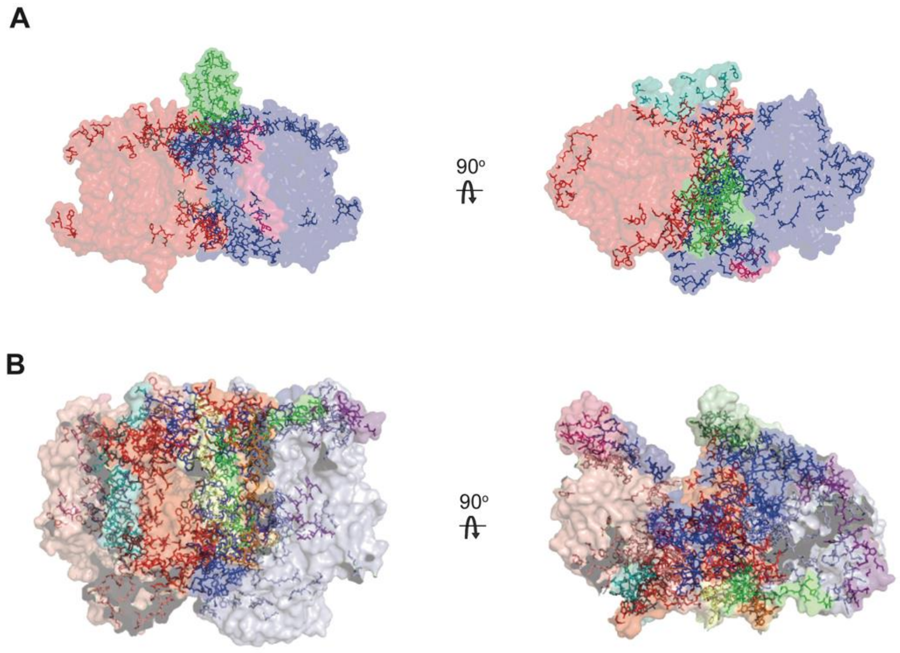

Supplementary Figure S5. Protein structures showing inter-subunit interface residues in the plastid-encoded photosystem proteins. Only proteins analysed in this study are shown. Residues identified at inter-subunit interfaces shown as sticks. **A**) Analysis of the 5L8R structure of photosystem I. Red, PsaA; blue, PsaB; green, PsaC; pink, PsaI and teal, PsaJ. **B**) Analysis of the 7OUI structure of photosystem II. Red, PsbA; light blue, PsbB; salmon, PsbC; blue, PsbD; pale green, PsbE; dark green, PsbF; purple, PsbH; teal, PsbI; dark blue, PsbK; green, PsbL; orange, PsbM; pale yellow, PsbT and pink, PsbZ.
