# Supplementary material for: Widespread adaptive evolution in angiosperm photosystems provides insight into the evolution of photosystem II repair
Source: Plant Cell. 2024 Oct 15;37(1):koae281. doi: 10.1093/plcell/koae281 (PMC11663578; doi:10.1093/plcell/koae281)
Supplement: koae281_Supplementary_Data [file tpc.00578.2024-s11.pdf › tpc.00578.2024-s11.pdf]

THE PLANT CELL

Widespread adaptive evolution in the photosystems of angiosperms provides insight into the evolution of photosystem II repair.

Elizabeth Robbins and Steven Kelly

Review Timeline:

Submission Date:	12-Jun-2024
Editorial Decision:	15-Aug-2024
Revision Received:	16-Sep-2024
Accepted:	19-Sep-2024

Prof. Steven Kelly
University of Oxford
Oxford
United Kingdom

Dear Dr. Kelly:

We have received reviews of your manuscript entitled "Widespread adaptive evolution in the photosystems of angiosperms provides new insight into the evolution of photosystem II repair." Thank you for submitting your best work to The Plant Cell. The editorial board agrees that the work you describe is substantive, falls within the scope of the journal, and may become acceptable for publication, pending revision and potential re-review.

I share the enthusiasm of all three reviewers! However, it will be important for you to fully address some of the reviewers concerns. We would also like to see some discussion in the Introduction about why you have limited your analysis to angiosperms and some thoughts on what you might see in other Archaeplastida lineages could also be included in the Discussion. As you will see, each of the reviewers have some concerns about the strength of assertions based completely on the patterns you are seeing in your comparative analyses. Please discuss the assumptions and limitations of your models. You may want to frame your conclusions as hypotheses that should be tested with genome editing experiments in tractable study systems. Finally, I strongly agree with point #7 raised by Reviewer #3. Please put your alignments and trees into a stable data package with a doi (e.g., a zenodo repository). I should stress that we are reluctant to see manuscripts undergoing multiple rounds of revision and would be unlikely to offer you more than one chance to satisfy the reviewers.

We think the work described in this manuscript would be well suited for our Phylogenomics Focus Issue (See https://academic.oup.com/plcell/pages/the-plant-cell-call-for-papers#focus%20on%20phylogenomics). If accepted, the manuscript would be published on-line and added to the Phylogenomics Focus Issue page when it is launched by next summer. You may be able to add the tag for this Focus Issue during resubmission.

Please contact us if there are ambiguous comments or if you wish to discuss the revision.

Given the nature of the comments, we are offering you 60 days from when we have issued this decision to complete the revision. If a revision is not returned within this time frame, and if you have not been granted an extension, we will withdraw the manuscript, which will leave you free to submit the work elsewhere. If you need an extension, we encourage you to contact us at any point before the 60 days have passed.

When you are ready to submit the revised version, please upload a highlighted copy that indicates all changes made in response to the editor and reviewer recommendations. Include an itemized list of all changes made in response to each of the reviewer's suggestions in the "Response to Reviewers" section; please note that reviewers do not have access to your cover letter, nor was this decision letter shared with them.

Thank you for the privilege of reviewing your work. We look forward to receiving your revised manuscript.

Sincerely,

The Plant Cell Editorial Board

Please note the following:

-The Plant Cell now requires authors to complete and submit an author revisions checklist upon submission of a revised manuscript. The aim of the checklist is to aid authors in preparing a high-quality manuscript, facilitate the review and assessment of revised manuscripts, and help to ensure that journal standards are maintained across the board. If your manuscript is accepted, the completed checklist will be published as supplemental material attached to the article online. Please download a copy of the checklist (pdf fillable form) at this link, for submission with your revised manuscript: https://tpc.msubmit.net/html/Author_Revisions_Checklist.pdf.

-Supplemental materials should be restricted to large datasets and tables, presentation of replicates, and validation of reagents, methods, or genotypes. Any data that are used to support the major claims must be in the main manuscript. Supplemental figure legends must indicate what figure in the main manuscript is supported by the supplemental data

presented. Please justify how each of the supplemental figures meet the criteria.

-Sampling methods and nature of "biological replicates" should be described precisely (i.e. different plants, parts of plants, pooled tissue, independent pools of tissue, sampled at different times, etc), along with a clear description of and rationale for any statistical analyses conducted. The reader should know exactly what was sampled; what forms the basis of the calculation of any means and statistical parameters reported. This is also necessary to ensure that proper statistical analysis was conducted.

-Want to add this revision deadline to your calendar? Click below!

--- Reviewer comments:

Reviewer #1 (Comments for the Author):

Robbins and Kelly presented an interesting computational study on the evolution of photosystems in angiosperms. The authors show that about 7% of residues in all photosystem sequences encoded in the plastid genomes feature signatures of adaptive evolution. The authors suggest that some of the observed sites with signatures of evolution favour substitutions that destabilise D1, which could facilitate turn-over and repair.

This is an exciting piece of research, and the paper is clear and well written. Materials and methods are well described, and the conclusions are reasonably supported by the proposed data. While the authors state that some of the identified mutations "reduce the energetic barrier for D1 turn-over and photosystem repair", or that "12 substitutions significantly destabilised their respective inter-subunit interface" ... Given that these observations are based on calculations, I think they should be presented more as hypotheses and/or predictions rather than as statements of fact. At least until further experimental validation is presented by them or others to show that such is indeed the case.

Finally, while your focus is solely on angiosperms, I would like you to provide a bit of a bigger picture view in your discussion:

Why would photosystems in angiosperms be evolving in this direction?

Are all angiosperm lineages evolving similarly or are the various observations that you made clustered in some lineages in particular? I would imagine only some groups might show this, but if it is an observation applied to most angiosperms what are the implications of this?

Would it be expected that photosystems in other plant or algal lineages are also evolving in a similar way?

Also, I would like you to rationalise in your discussion how is it that "reducing the energetic barrier for D1 turn-over and photosystem repair" can lead to an adaptive advantage. One could argue that more D1 turnover and repair wouldn't be very economical and would lead to an increase in the overall energetic cost of maintaining active photosystem II, which may be considered disadvantageous. There should be some kind of trade off. Please discuss.

Minor points:

Line 36. No one has demonstrated that oxygenic photosynthesis originated ca. 3.0 billion years ago. There is evidence suggesting that oxygenic photosynthesis had already evolved by 3.0 billion years ago... but to be accurate, no one really knows when oxygenic photosynthesis originated.

Line 55-56. "which remains largely undamaged." Perhaps also cite this:
https://www.sciencedirect.com/science/article/pii/S0014579311008829

Based on this paper, there might be a gradient of damage, with D1 having the fastest turnover, but with D2 and CP43/CP47 also being the subject of damage/repair at considerably faster rates than PSI subunits.

Section starting with line 186. You state things like: "We found none of the 63 adaptive substitutions at cofactor binding sites significantly affected ligand binding (Supplementary Table S1)." However, it isn't clear how you get to this finding. I think you need to present the rationales for the energy calculations here in the main text and how you derive the conclusions you do. Just directing the reader to the supplementary information isn't sufficient. Also, explain the concept of "inter-subunit interaction energy" shown in Supplementary Figure S2.

Has anyone validated experimentally that the calculated energies do indeed translate to stability or instability on a molecular complex in other systems? If so, it would boost your hypothesis so you might want to cite and discuss

accordingly.

Line 309. In my experience with IQTREE, I've found that 1000 replicates are often not sufficient to reach convergence according to the software specifications. Can you confirm that it was indeed the case and that the tree inference ran to completion?

Lines 311-313. Please provide this tree. In addition, provide all relevant sequence alignments and ancestral sequence reconstruction (ASR) data. Do provide as supplementary data, but if this data is already publicly available and accessible via your other published paper, please say so.

Lines 322-328. It isn't clear how this was done. Did you prepare this manually from the ASR data? Did you create some code to extract this information? Can you provide the tabulation of the substitutions mentioned? Please be more detailed to enable reproducibility here.

Lines 331 and 332. Please, specify the organisms used for these structures and the resolution of the structures. The PSI structure used is rather old and may not be as complete as more recent ones, and it lacks some cofactors. For example, only 3 carotenoids were resolved, when PSI has many more than PSII. I would encourage you to try to find one of the more recent and higher resolution structures of PSI and check if you can improve/update some of your substitution assignments.

Reviewer #2 (Comments for the Author):

The paper by Robbins and Kelly examines the adaptive evolution in the photosystems of angiosperms and through this illustrate that a suite of changes with the evolution of the angiosperms has destabilised the interaction of photosystem II with the D1 subunit reducing the energetic barrier for D1 turn-over and photosystem repair. They hypothesise that these
substitutions result in less photodamage being required to initiate D1 removal and replacement. The MS is well written and easy to follow and explores a very interesting topic regarding D1 damage and repair and state-of-the-art methodology for exploring this area.
I greatly enjoyed reading the MS and only have a few questions for clarification and to emphasize the impact and potential translational aspects of the research.

It would be nice to see a little more information on the biological significance of more rapid D1 turnover on evolution of the angiosperms and the evolutionary advantage. And it would be nice to acknowledge how the information provided in the MS could be used for crop improvements etc.
What are the costs of increased D1 turnover relative to the benefit?
There are several sections of the discussion that are repetitive of the introduction.
Have the authors considered biologically testing the theory that evolution has led to a reduction in photodamage required to induce D1 replacement and repair?

Reviewer #3 (Comments for the Author):

This manuscript investigates signatures of selection on protein subunits of angiosperm photosystems based on phylogenetic analysis of protein-coding sequences and protein structural modeling. The authors identify a substantial number of putative sites that may have been subject to adaptive substitutions and propose that there has been selection to destabilize the binding interface of the D1 subunit. They posit that D1 destabilization may facilitate turnover of this subunit in response to photoinhibition. Overall, I found this to be a very clearly written and nicely illustrated manuscript. The analysis is substantial in scope (hundreds of angiosperm plastid genomes) and detailed in its description of the location of substitutions in a 3D structural context. I do have substantial concerns, however, that the lines of evidence in support of the stated conclusions are very tentative such that those conclusions should be taken with a great deal of caution and skepticism. My specific concerns are detailed in the comments below.

Major Comments:

1. One of the two lines of evidence supporting the conclusion that the identified substitutions are adaptive is that many of them were "recurrent," meaning that they occurred in parallel in two or more evolutionary lineages. I appreciate that the authors tested the frequency of recurrent substitutions against a null model to determine whether recurrent substitutions occurred more often than expected by chance, but I am concerned that their approach may not have adequately distinguished the ability of purifying selection to produce similar patterns. Specifically, they applied a

transition matrix (in this case, JTT) uniformly across the length of each protein. This matrix captures the effects of selection (and mutation) that make certain pairs of amino acids more or less "exchangeable." I agree that this exchangeability should be considered in generating null expectations, but it is a significant limitation to assume that transition probabilities are the same across all sites. Some sites can tolerate a specific type of substitution, while the exact same substitution is deleterious at a different site, which is why methods that employ position-specific information (e.g., HMMs) can be more sensitive in detecting homology than search methods that apply a uniform Q-matrix. Therefore, some recurrent mutations at a given site could reflect the fact that only a small number of amino acid states are tolerated at that site rather than selection is repeatedly favoring the observed substitution.

2. The second line of evidence for positive selection is the use of FUBAR to identify codons with dN/dS values > 1. This genre of selection tests is widely used and appropriate for analysis of phylogenetic datasets. I don't have any concerns with applying and reporting positive selection tests in this case, but caution is probably always necessary in interpreting results because these tests have been extensively criticized for risks of both false positives and false negatives. Therefore, I would tend disagree with the authors' conclusion that satisfying either of these criteria constitutes "robust evidence" of adaptive evolution (line 146). Even for the sites that satisfy both criteria, I would still consider the evidence tentative and indirect.

3. The authors emphasize in the abstract, the last section of the results, and the discussion that selection has favored destabilization of D1 (=PsbA) binding interfaces and speculate that this is an adaptation to facilitate easier turnover of the D1 subunit during PSII repair. This conclusion is based on the fact that all recurrent substitutions at D1 interfaces have a destabilizing effect. However, there were only five recurrent substitutions, and one of them had essentially no effect (Figure S2). Given that the authors found a relatively even split between stabilizing and destabilizing, I don't feel that the pattern observed in these five (four) sites to be very compelling, especially because it appears to be a post hoc interpretation after looking at all subunits (which makes it problematic to pull out this one set of sites and report an uncorrected P-value for a one-tailed test; line 195). Therefore, although the authors are putting forward a very interesting and clearly articulated idea, there seems to be limited evidence for it at this point. I feel the conclusions are overstated in multiple places relative to the strength of the supporting evidence.

4. I believe the (de)stabilization effects of amino acid substitutions were all measured relative to a single reference complex from an extant species. But the actual substitutions of interest occurred in specific lineages that would have differed from this reference at multiple sites. Therefore, this approach does not consider the possibility of epistatic interactions between substitutions with respect to effects on binding energy. Testing the predicted effects of substitutions in the reconstructed ancestral sequence background on which they were inferred to occur could assess whether there is a pattern of coevolutionary responses to changes at other sites.

5. I did not see any consideration of C-to-U RNA editing sites in plastid genes. These are not especially abundant in angiosperm plastid genomes, but there are some in photosystem genes. C-to-T nucleotide substitutions at such sites are common and could be mistaken as recurrent changes in amino acid sequence even though they are effectively silent at the protein level.

6. The authors did not find effects on predicted ligand binding affinity from any observed recurrent substitutions. Did this estimate of binding energy have the ability to detect such changes? I would consider testing randomly introduced substitutions at ligand binding sites to determine what fraction of them have a predicted effect on binding.

7. The manuscript states that all data are provided in the supplementary material, but I am not seeing access to sequence alignments or trees. Unless I am missing those somewhere, I would suggest making all alignments (trimmed and untrimmed) and inferred tree(s) available in a data repository. The authors' nice efforts in assembling these analyses could be valuable to other researchers.

Minor Comments

8. Would "enriched" and "depleted" be preferable usage to "over-enriched" and "under-enriched"? Or perhaps overrepresented and underrepresented?

9. Figure S2 is presented as a box plot even though it is a distribution of only 5 points. I think it would be more transparent to simply show the five points. In my view, a boxplot is appropriate for sample sizes that are large enough to break into quartiles in a meaningful way.

10. Line 312. The authors use the term "basal angiosperm." I know that this usage is fairly common in the literature, but I would argue that it's misleading when referring to extant taxa that are not actually basal in the tree (see Crisp and Cook 2005 for discussion of this issue; doi: 10.1016/j.tree.2004.11.010). I would suggest "... as they are the sister lineage to rest of the sampled angiosperms in the dataset."

Editor comments
Dear Dr. Kelly:
We have received reviews of your manuscript entitled "Widespread adaptive evolution in the photosystems of angiosperms provides new insight into the evolution of photosystem II repair." Thank you for submitting your best work to The Plant Cell. The editorial board agrees that the work you describe is substantive, falls within the scope of the journal, and may become acceptable for publication, pending revision and potential re-review.
I share the enthusiasm of all three reviewers! However, it will be important for you to fully address some of the reviewers concerns. We would also like to see some discussion in the Introduction about why you have limited your analysis to angiosperms and some thoughts on what you might see in other Archaeplastida lineages could also be included in the Discussion. As you will see, each of the reviewers have some concerns about the strength of assertions based completely on the patterns you are seeing in your comparative analyses. Please discuss the assumptions and limitations of your models. You may want to frame your conclusions as hypotheses that should be tested with genome editing experiments in tractable study systems. Finally, I strongly agree with point #7 raised by Reviewer #3. Please put your alignments and trees into a stable data package with a doi (e.g., a zenodo repository). I should stress that we are reluctant to see manuscripts undergoing multiple rounds of revision and would be unlikely to offer you more than one chance to satisfy the reviewers.
We think the work described in this manuscript would be well suited for our Phylogenomics Focus Issue (See https://academic.oup.com/plcell/pages/the-plant-cell-call-for-papers#focus%20on%20phylogenomics). If accepted, the manuscript would be published on-line and added to the Phylogenomics Focus Issue page when it is launched by next summer. You may be able to add the tag for this Focus Issue during resubmission.
Please contact us if there are ambiguous comments or if you wish to discuss the revision. Given the nature of the comments, we are offering you 60 days from when we have issued this decision to complete the revision. If a revision is not returned within this time frame, and if you have not been granted an extension, we will withdraw the manuscript, which will leave you free to submit the work elsewhere. If you need an extension, we encourage you to contact us at any point before the 60 days have passed.
When you are ready to submit the revised version, please upload a highlighted copy that indicates all changes made in response to the editor and reviewer recommendations. Include an itemized list of all changes made in response to each of the reviewer's suggestions in the "Response to Reviewers" section; please note that reviewers do not have access to your cover letter, nor was this decision letter shared with them.
Thank you for the privilege of reviewing your work. We look forward to receiving your revised manuscript.
Sincerely,

The Plant Cell Board of Editors

Response to editor comments
We are very grateful to the editors and expert reviewers for their critical evaluation and positive assessment of our work. In the revised manuscript we have addressed all of the comments and questions that were raised by the reviewers and provided a Zenodo archive that contains all of the data related to the analyses presented in the manuscript. The details of the changes that were made are described in full in the accompanying point-by-point responses below. We hope that you will find our revised manuscript suitable for publication in Plant Cell.

Regarding the additional discussion that is requested in the introduction. A paragraph on why we chose to investigate angiosperms was included in the discussion section of the original manuscript. The paragraph in question is the second paragraph of the discussion where it reads:

“The analysis presented here focused on angiosperms, and not cyanobacteria or algae, for several reasons. First, the angiosperm clade contains the majority of crop plants, including food plants for direct human consumption, animal feed, biofuels, textiles, building materials and pharmaceuticals (Raskin, et al. 2002; Leff, et al. 2004; Mahapatra, et al. 2021). Consequently, understanding the evolution of their photosynthetic apparatus, i.e., what evolution has tried and tested, holds most potential to inform future crop improvement strategies. Second, a large number of angiosperm plastid genomes have been sequenced allowing for powerful phylogenetic analyses (Christenhusz and Byng 2016). Third, the high degree of gene conservation in the plastid genome of angiosperms enabled a robust phylogenetic tree to be constructed from which adaptive evolution was inferred. The analysis presented here will provide a foundation and point of comparison for investigation of adaptive evolution in photosystems of other lineages, such as algae or cyanobacteria, in future studies.”

We have moved the “why angiosperms part of this paragraph to the introduction” but are happy to move it back to the discussion if the editors or reviewers preferred its original location. We have expanded the “what do we expect to see in other lineages” section of the discussion. The new paragraph is provided below:

“The analysis presented here focused on angiosperms as information gained here holds most potential to inform future crop improvement strategies. However, the analysis presented here will provide a foundation and point of comparison for investigation of adaptive evolution in photosystems of other lineages, such as algae or cyanobacteria, in future studies. In this context it is interesting to note that there are differences in the repertoire of genes that constitute the photosystems in these lineages. For example, the D1 gene is encoded by a multi-gene family in cyanobacteria (Mulo, et al. 2009). Here, different family members are differentially expressed according to environmental cues to adapt photochemical fitness to solar intensity (Vinyard, et al. 2013). Given the sub-functionalisation that has occurred within this gene family, it may be that different gene family members are adapting along different evolutionary trajectories. For example, the high light adapted isoform may exhibit an analogous trajectory as described here for angiosperm, reducing the energetic barrier for repair, while the low light adapted version exhibits a different trend. Furthermore, comparison between different Archaeplastida lineages may provide further insight into global trends not detectable through analysis of angiosperms alone.”

Reviewer #1 (Comments for the Author):

Comment 1

Robbins and Kelly presented an interesting computational study on the evolution of photosystems in angiosperms. The authors show that about 7% of residues in all photosystem sequences encoded in the plastid genomes feature signatures of adaptive evolution. The authors suggest that some of the observed sites with signatures of evolution favour substitutions that destabilise D1, which could facilitate turn-over and repair.

This is an exciting piece of research, and the paper is clear and well written. Materials and methods are well described, and the conclusions are reasonably supported by the proposed data. While the authors state that some of the identified mutations "reduce the energetic barrier for D1 turn-over and photosystem repair", or that "12 substitutions significantly destabilised their respective inter-subunit interface" ... Given that these observations are based on calculations, I think they should be presented more as hypotheses and/or predictions rather than as statements of fact. At least until further experimental validation is presented by them or others to show that such is indeed the case.

Response 1

We thank the reviewer for their enthusiasm for our study. We agree that our phylogenetic study has produced interesting hypotheses that require experimental validation. The manuscript has been changed throughout to make this clearer.

Comment 2

Finally, while your focus is solely on angiosperms, I would like you to provide a bit of a bigger picture view in your discussion: Why would photosystems in angiosperms be evolving in this direction? Are all angiosperm lineages evolving similarly or are the various observations that you made clustered in some lineages in particular? I would imagine only some groups might show this, but if it is an observation applied to most angiosperms what are the implications of this? Would it be expected that photosystems in other plant or algal lineages are also evolving in a similar way?

Response 2:
We thank the reviewer for this suggestion. We have added substantially to the discussion section of the manuscript on the bigger picture as well as what may be happening in other lineages (as outlined in the response to the comment below, and in response to comments made by the editor and other reviewers). As requested, we have also added additional discussion of the distribution of the changes we have observed across the phylogeny. We have also noted that it will be interesting to perform a large-scale analysis of photoinhibition on species within these groups to understand how these changes have altered the function of the photosystems. However, such a study is beyond the scope of the comparative genomic investigation presented here.

The additional paragraph is copied below:

"The recurrent substitutions identified in this study are not uniformly distributed across the phylogeny. For example, E235A in D1 was identified in 15 different families (Cornales, Arecales, Laurales, Chloranthales, Nymphaeales, Gentianales, Rosales, Poales, Ericales, Malvales, Caryophyllales, Zingiberales, Myrtales, Huerteales, and Vitales) while E243G in D1 was only observed in four families (Brassicales, Apiales, Rosales, and Ericales). These differences in distribution may allow the effect of these substitutions to be experimentally distinguished. In this context, it will be interesting to perform a large-scale analysis of photoinhibition on species within these groups to understand how these changes have altered the function of the photosystems. Specifically, it will be interesting to determine if species harbouring these changes are less subject to photoinhibition across a range of different light intensities. Moreover, it will also be interesting to determine if introduction of these changes into species in which they have not evolved can result in a reduction in photoinhibition."

Comment 3
Also, I would like you to rationalise in your discussion how is it that "reducing the energetic barrier for D1 turn-over and photosystem repair" can lead to an adaptive advantage. One could argue that more D1 turnover and repair wouldn't be very economical and would lead to an increase in the overall energetic cost of maintaining active photosystem II, which may be considered disadvantageous. There should be some kind of trade off. Please discuss.

Response 3
We thank the reviewer for raising this interesting question. We have added a paragraph in the discussion to address this directly and it is copied below. In brief, repair of photoinhibited photosystem II is triggered by detection of damaged D1. Functional photosystems are not turned over in the absence of detection of D1 damage. Thus, altering the energetic barrier to D1 repair would have no effect on photosynthesis or energetic cost in the absence of D1 damage. This is because all photosystems are active and thus the cost to the cell would be the same irrespective any change in the energetic barrier for D1 replacement. However, in the presence of D1 damage reducing the energetic barrier to D1 replacement would speed up the rate at which damaged photosystems can be repaired. Thus, the effect of reducing the energetic barrier to D1 repair, and consequently speeding up the rate of photosystem repair, would mean that at any given time the proportion of damaged photosystems in a cell would be decreased. This in turn would increase the conversion of incident light energy into chemical energy and growth, thereby conferring a selective advantage.

The new discussion paragraph is copied below.

“The question arises as to why reducing the energetic barrier for D1 turn-over and photosystem II repair would lead to an adaptive advantage. At any given time, the proportion of damaged photosystems is a function of the incident light, the activity of photoprotective mechanisms, and the rate of photosystem repair (Su, et al. 2023). Each of these factors interact to alter the proportion of photosystem II containing a non-functional D1 subunit and are thus in a non-productive photoinhibited state. Evolution by natural selection can act to modulate each of these factors to maximise the conversion of incident light energy into chemical energy and growth. As turn-over is triggered by the detection of non-functional D1, reducing the energetic barrier for D1 removal and replacement would in turn increase the rate at which damaged photosystem II complexes can be repaired. Furthermore, as photoinhibition only occurs when the rate of repair is unable to keep up with the rate of damage (Prasil 1992), the overall effect of reducing the energetic barrier to repair would be to have a lower proportion of non-functional photosystem II complexes, thereby increasing the conversion of incident light energy into chemical energy and growth.”

Comment 4
Line 36. No one has demonstrated that oxygenic photosynthesis originated ca. 3.0 billion years ago. There is evidence suggesting that oxygenic photosynthesis had already evolved by 3.0 billion years ago... but to be accurate, no one really knows when oxygenic photosynthesis originated.

Response 4
We thank the reviewer for spotting this mistake. The introduction has been amended.

Comment 5
Line 55-56. "which remains largely undamaged." Perhaps also cite this: https://www.sciencedirect.com/science/article/pii/S0014579311008829. Based on this paper, there might be a gradient of damage, with D1 having the fastest turnover, but with D2 and CP43/CP47 also being the subject of damage/repair at considerably faster rates than PSI subunits.

Response 5
We thank the reviewer for their suggestion. The citation has been added and sentence rephrased to reflect that D1 being a hotspot of damage reduces the burden of damage on the remainder of the PSII complex.

Comment 6
Section starting with line 186. You state things like: "We found none of the 63 adaptive substitutions at cofactor binding sites significantly affected ligand binding (Supplementary Table S1)." However, it isn't clear how you get to this finding. I think you need to present the rationales for the energy calculations here in the main text and how you derive the conclusions you do. Just directing the reader to the supplementary information isn't sufficient. Also, explain the concept of "inter-subunit interaction energy" shown in Supplementary Figure S2.

Response 6
We thank the reviewer for their comment. For clarity, we have added an explanation in this paragraph explaining that the change in protein-cofactor and protein-protein interactions are calculated from alterations in structural features upon substitution which have subsequent impacts on empirical energy calculations of the respective interface.

Comment 7
Has anyone validated experimentally that the calculated energies do indeed translate to stability or instability on a molecular complex in other systems? If so, it would boost your hypothesis so you might want to cite and discuss accordingly.

Response 7
We thank the reviewer for this suggestion. Several studies have used computational predictions of stability altering substitutions to alter the stability of protein sequences. For example, there have been successful efforts in improving the stability of haloalkane dehalogenase (Floor, et al. 2014),

pyruvate formate-lyase (Yang, et al. 2007), cutinase (Shirke, et al. 2016), acetylcholinesterase (Goldenzweig, et al. 2016) and methionine aminopeptidase (Borgo, et al. 2012). The methods have also been tested across a range of stability altering mutations in large scale meta-analyses of different methods have also been performed (Marabotti, et al. 2021). We have followed recommendations from these meta-analyses.

We have added the following sentences to the revised discussion to address this point:

"Computational predictions of structural stability have been used repeatedly to inform the rational design and improvement of many proteins, for example (Yang, et al. 2007; Floor, et al. 2014; Goldenzweig, et al. 2016; Shirke, et al. 2016). However, these methods are not yet perfect (Marabotti, et al. 2021) and future re-evaluation of the data presented here with more sophisticated methods may uncover additional changes to the photosystems in angiosperms not reported here."

Comment 8
Line 309. In my experience with IQTREE, I've found that 1000 replicates are often not sufficient to reach convergence according to the software specifications. Can you confirm that it was indeed the case and that the tree inference ran to completion?

Response 8
We agree with the reviewer here and it is always important to check this. In our analysis, we were careful to check that convergence had been reached. The bootstrap correlation coefficient of split occurrence frequencies reached a value of 0.991 by iteration 100, 0.998 by iteration number 200, and the tree search completed at iteration number 272. The log file returned by IQTREE for the tree inference is now provided in the Zenodo archive.

Comment 9
Lines 311-313. Please provide this tree. In addition, provide all relevant sequence alignments and ancestral sequence reconstruction (ASR) data. Do provide as supplementary data, but if this data is already publicly available and accessible via your other published paper, please say so.

Response 9
The requested data was too large to include as a supplemental file. However, to get around this constraint we have created a Zenodo archive that contains the species tree as well as the alignment files and ancestral state reconstructions. A link to the Zenodo archive is provided in the revised manuscript.

Comment 10
Lines 322-328. It isn't clear how this was done. Did you prepare this manually from the ASR data? Did you create some code to extract this information? Can you provide the tabulation of the substitutions mentioned? Please be more detailed to enable reproducibility here.

Response 10.
This was done manually using Python scripts directly from the ancestral state reconstruction data, this has been stated more clearly in the methods. All substitutions identified have now been provided in the revised Supplementary Table S3. We have also provided the code that counts the substitutions in a GitHub repository https://github.com/ElizabethRobbins/Identify_amino_acids_substitutions_in_phylogeny.git that is included in the revised manuscript.

Comment 11
Lines 331 and 332. Please, specify the organisms used for these structures and the resolution of the structures. The PSI structure used is rather old and may not be as complete as more recent ones, and it lacks some cofactors. For example, only 3 carotenoids were resolved, when PSI has many more than PSII. I would encourage you to try to find one of the more recent and higher resolution structures of PSI and check if you can improve/update some of your substitution assignments.

Response 11
We thank the reviewer for their comment. The species and resolution of the structures used have been added to the methods. The analysis has also been completely redone with the 5L8R PSI structure obtained from pea in 2017. Although some of the numbers have changed, changing the structure did not alter any of the conclusions made in the original manuscript. We think that the inclusion of this additional analysis has strengthened the manuscript.

Reviewer #2 (Comments for the Author):

Comment 1
The paper by Robbins and Kelly examines the adaptive evolution in the photosystems of angiosperms and through this illustrate that a suite of changes with the evolution of the angiosperms has destabilised the interaction of photosystem II with the D1 subunit reducing the energetic barrier for D1 turn-over and photosystem repair. They hypothesise that these substitutions result in less photodamage being required to initiate D1 removal and replacement. The MS is well written and easy to follow and explores a very interesting topic regarding D1 damage and repair and state-of-the-art methodology for exploring this area.

Response 1
We thank the reviewer for their supportive comments.

Comment 2
I greatly enjoyed reading the MS and only have a few questions for clarification and to emphasize the impact and potential translational aspects of the research. It would be nice to see a little more information on the biological significance of more rapid D1 turnover on evolution of the angiosperms and the evolutionary advantage. And it would be nice to acknowledge how the information provided in the MS could be used for crop improvements etc. What are the costs of increased D1 turnover relative to the benefit?

Response 2
We thank the reviewer again for their supportive comments. A paragraph on the evolutionary advantage and an additional paragraph on crop improvement opportunities have been added to the discussion and are copied below.

"The question arises as to why reducing the energetic barrier for D1 turn-over and photosystem II repair would lead to an adaptive advantage. At any given time, the proportion of damaged photosystems is a function of the incident light, the activity of photoprotective mechanisms, and the rate of photosystem repair (Su, et al. 2023). Each of these factors interact to alter the proportion of photosystem II containing a non-functional D1 subunit and are thus in a non-productive photoinhibited state. Evolution by natural selection can act to modulate each of these factors to maximise the conversion of incident light energy into chemical energy and growth. As turn-over is triggered by the detection of non-functional D1, reducing the energetic barrier for D1 removal and replacement would in turn increase the rate at which damaged photosystem II complexes can be repaired. Furthermore, as photoinhibition only occurs when the rate of repair is unable to keep up with the rate of damage (Prasil 1992), the overall effect of reducing the energetic barrier to repair would be to have a lower proportion of non-functional photosystem II complexes, thereby increasing the conversion of incident light energy into chemical energy and growth.

The recurrent substitutions identified in this study are not uniformly distributed across the phylogeny. For example, E235A in D1 was identified in 15 different families (Cornales, Arecales, Laurales, Chloranthales, Nymphaeales, Gentianales, Rosales, Poales, Ericales, Malvales, Caryophyllales, Zingiberales, Myrtales, Huerteales, and Vitales) while E243G in D1 was only observed in four families (Brassicales, Apiales, Rosales, and Ericales). These differences in distribution may allow the effect of these substitutions to be experimentally distinguished. In this context, it will be interesting to perform a large-scale analysis of photoinhibition on species within these groups to understand how these changes have altered the function of the photosystems.

Specifically, it will be interesting to determine if species harbouring these changes are less subject to photoinhibition across a range of different light intensities. Moreover, it will also be interesting to determine if introduction of these changes into species in which they have not evolved can result in a reduction in photoinhibition."

Comment 3
There are several sections of the discussion that are repetitive of the introduction.

Response 3
We appreciate the reviewers concern here. We have reduced the overlap between the introduction and the discussion.

Comment 4
Have the authors considered biologically testing the theory that evolution has led to a reduction in photodamage required to induce D1 replacement and repair?

Response 4
We thank the reviewer for their comment. We agree it will be very interesting (and potentially very useful) to test the hypotheses generated in this study. We are actively pursuing this, but plastid transformation is difficult and genome editing in the plastid has only been achieved by a very small number of people worldwide. We hope to have results of these experimental investigations within the next 4-8 years. Accordingly, we feel that this request is outside the scope of this comparative genomic investigation. We hope that, in addition to our own efforts, our analyses will inspire many others to test the findings that are presented in this work.

Reviewer #3 (Comments for the Author):
Comment 1
This manuscript investigates signatures of selection on protein subunits of angiosperm photosystems based on phylogenetic analysis of protein-coding sequences and protein structural modelling. The authors identify a substantial number of putative sites that may have been subject to adaptive substitutions and propose that there has been selection to destabilize the binding interface of the D1 subunit. They posit that D1 destabilization may facilitate turnover of this subunit in response to photoinhibition. Overall, I found this to be a very clearly written and nicely illustrated manuscript. The analysis is substantial in scope (hundreds of angiosperm plastid genomes) and detailed in its description of the location of substitutions in a 3D structural context.

Response 1
We thank the reviewer for their supportive comments.

Comment 2
I do have substantial concerns, however, that the lines of evidence in support of the stated conclusions are very tentative such that those conclusions should be taken with a great deal of caution and scepticism. My specific concerns are detailed in the comments below. One of the two lines of evidence supporting the conclusion that the identified substitutions are adaptive is that many of them were "recurrent," meaning that they occurred in parallel in two or more evolutionary lineages. I appreciate that the authors tested the frequency of recurrent substitutions against a null model to determine whether recurrent substitutions occurred more often than expected by chance, but I am concerned that their approach may not have adequately distinguished the ability of purifying selection to produce similar patterns. Specifically, they applied a transition matrix (in this case, JTT) uniformly across the length of each protein. This matrix captures the effects of selection (and mutation) that make certain pairs of amino acids more or less "exchangeable." I agree that this exchangeability should be considered in generating null expectations, but it is a significant limitation to assume that transition probabilities are the same across all sites. Some sites can tolerate a specific type of substitution, while the exact same substitution is deleterious at a different site, which is why methods that employ position-specific information (e.g., HMMs) can be more sensitive in detecting homology than search methods that apply a uniform Q-matrix. Therefore, some recurrent mutations at a given site could reflect the fact that only a small number of amino

acid states are tolerated at that site rather than selection is repeatedly favouring the observed substitution.

Response 2

We thank the reviewer for the detailed and thoughtful analysis of our approach. During the development of our approach we also considered the potential limitations, particularly the uniform distribution across sites. We also considered the use of HMMs to overcome this limitation, as suggested by the reviewer, but discounted this as a solution for two principle reasons. First, preserving the relationship between the sequences by simulating the sequences on a tree is critical for correctly controlling for the underlying relationship and evolutionary distance between the sequences that are sampled in the study. Sequences simulated using HMMs cannot capture the phylogenetic non-independence of the sequences nor the evolutionary distance between the sequences and thus cannot achieve the necessary control for the inter-relationship and extent of diversity that is present in the dataset being interrogated. Second, simulating sets of sequences using a HMM returns cohorts of sequences with the same amino acid distribution as in the input alignments. This approach therefore results in overfitting such that the site distributions in the simulated alignments are the same as the site distributions in the dataset being interrogated. Thus, for these two reasons we did not use HMMs to generate the sequences. We agree that the model, like all models in phylogenetic methods, can be improved in the future to better account for rate variability between sites and we are actively working on this. We are also working to prepare a standalone software tool that researchers can use to conduct their own analyses of substitution recurrence. We hope to be able to post a preprint on this software within the next year.

We also agree with the reviewer that there is a possibility that some recurrent sites could be caused by purifying selection. We have now conducted an analysis of the co-occurrence of recurrent substitutions and purifying selection. This has revealed that there is a significant depletion of recurrent substitutions at sites that are detected as evolving under purifying selection. In combination with the results presented in the original manuscript, where we showed that there was a significant enrichment of recurrent substitutions at sites that are detected as evolving under positive selection, this provides evidence that the recurrent substitutions are not being caused by purifying selection. We have added the analysis of purifying selection to the revised manuscript and thank the reviewer for this suggestion.

Comment 3

The second line of evidence for positive selection is the use of FUBAR to identify codons with dN/dS values > 1. This genre of selection tests is widely used and appropriate for analysis of phylogenetic datasets. I don't have any concerns with applying and reporting positive selection tests in this case, but caution is probably always necessary in interpreting results because these tests have been extensively criticized for risks of both false positives and false negatives. Therefore, I would tend disagree with the authors' conclusion that satisfying either of these criteria constitutes "robust evidence" of adaptive evolution (line 146). Even for the sites that satisfy both criteria, I would still consider the evidence tentative and indirect.

Response 3

We appreciate the reviewers concern here. We have removed the word "robust" and altered the manuscript to say that both the positive selection sites and recurrently evolved sites may provide evidence of adaptive evolution.

Comment 4

The authors emphasize in the abstract, the last section of the results, and the discussion thatpur selection has favoured destabilization of D1 (=PsbA) binding interfaces and speculate that this is an adaptation to facilitate easier turnover of the D1 subunit during PSII repair. This conclusion is based on the fact that all recurrent substitutions at D1 interfaces have a destabilizing effect. However, there were only five recurrent substitutions, and one of them had essentially no effect (Figure S2). Given that the authors found a relatively even split between stabilizing and destabilizing, I don't feel that the pattern observed in these five (four) sites to be very compelling, especially because it appears to be a post hoc interpretation after looking at all subunits (which

makes it problematic to pull out this one set of sites and report an uncorrected P-value for a one-tailed test; line 195). Therefore, although the authors are putting forward a very interesting and clearly articulated idea, there seems to be limited evidence for it at this point. I feel the conclusions are overstated in multiple places relative to the strength of the supporting evidence.

Response 4
We thank the reviewer for their comments here. The purpose of this study was to analyse the molecular evolution of the photosystems to uncover evolutionary changes that would be of interest to the photosynthesis community. We fully appreciate that the results we have put forward in this manuscript are computational predictions and need experimental validation. In the revised manuscript we have toned down the language to make it clearer that these are computational predictions and need experimental testing. We feel the statistical test is valid as we are asking the very specific question of whether this set of substitutions destabilise the interaction of D1 with the core of the photosystem. We are not asking a more general question concerning the complete set of substitutions (categorised by interface or some other categorical variable) which would require p-value correction.

Comment 5
I believe the (de)stabilization effects of amino acid substitutions were all measured relative to a single reference complex from an extant species. But the actual substitutions of interest occurred in specific lineages that would have differed from this reference at multiple sites. Therefore, this approach does not consider the possibility of epistatic interactions between substitutions with respect to effects on binding energy. Testing the predicted effects of substitutions in the reconstructed ancestral sequence background on which they were inferred to occur could assess whether there is a pattern of coevolutionary responses to changes at other sites.

Response 5
The reviewer raises an interesting point here. There are a limited number of structures available on which to perform this analysis and structures for ancestral sequences are not available. One of our aims in conducting this analysis was to identify substitutions that could be introduced into extant species to test their effect in subsequent *in vivo* studies. This, combined with the limited availability of structures, guided our experimental design. In the future it may be possible to gain accurate models for these ancestral sequences, and in that case a follow-on study that re-evaluates the data presented in this work in the context of these newly generated structures may reveal additional insight to the results that we have presented here. However, even with the recent advances in AlphaFold and other structure prediction methods, the technology does not yet exist to be able to do this, and accordingly it is beyond the scope of the current study. What was reassuring was that we have performed a complete repeat of our analysis with a different structure in response reviewer 2 comment 11. This re-analysis did not change any of the conclusions presented in the manuscript.

Comment 6
I did not see any consideration of C-to-U RNA editing sites in plastid genes. These are not especially abundant in angiosperm plastid genomes, but there are some in photosystem genes. C-to-T nucleotide substitutions at such sites are common and could be mistaken as recurrent changes in amino acid sequence even though they are effectively silent at the protein level.

Response 6
The reviewers intuition is correct. We investigated RNA editing in great detail and the extent of the analysis and findings precluded their inclusion in this manuscript. We have prepared a separate manuscript on the evolution or RNA editing sites across the plastid genome in angiosperms. This manuscript includes all genes resident in the plastid genome (e.g. the transcription/translation machinery and other proteins involved in energy metabolism) and focusses on the evolution of RNA editing, rather than the evolution of the mechanisms of the encoded genes. Accordingly, this separate analysis does not fit with the work presented here. This separate manuscript is nearly ready for submission as a pre-print and we hope the reviewer will enjoy this work when it emerges.

Comment 7
The authors did not find effects on predicted ligand binding affinity from any observed recurrent substitutions. Did this estimate of binding energy have the ability to detect such changes? I would consider testing randomly introduced substitutions at ligand binding sites to determine what fraction of them have a predicted effect on binding.

Response 7
We appreciate the reviewers concern here. Yes, the estimate of binding energy did have the ability to detect such changes. The histogram below shows the results of complete mutational scanning of all possible changes in binding energy for all co-factor binding sites in photosystem I and photosystem II. Here, all 19 different amino acids were substituted at each cofactor binding site and the change in binding energy recorded. We have marked the largest stabilising and destabilising change obtained by a recurrent substitution as blue and red dashed lines, respectively. The observation that recurrent changes do not substantially affect co-factor binding is consistent with our finding that there is a depletion of recurrent substitutions at co-factor binding sites.

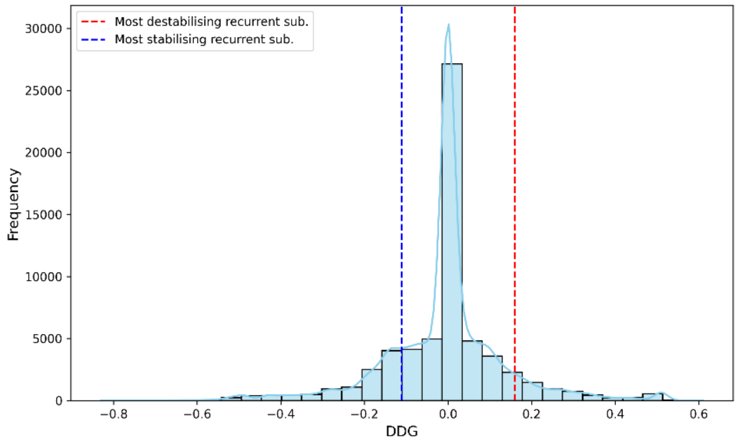

Comment 8
The manuscript states that all data are provided in the supplementary material, but I am not seeing access to sequence alignments or trees. Unless I am missing those somewhere, I would suggest making all alignments (trimmed and untrimmed) and inferred tree(s) available in a data repository. The authors' nice efforts in assembling these analyses could be valuable to other researchers.

Response 8
We thank the reviewer for their appreciation of the dataset. In the original manuscript we had provided all the result data in the supplemental material as the input data was too large to include as a supplemental file. However, we appreciate the reviewers request and we have now made the input datasets available as a Zenodo archive.

Comment 9
Would "enriched" and "depleted" be preferable usage to "over-enriched" and "under-enriched"? Or perhaps overrepresented and underrepresented?

Response 9
Thank you for your suggestion. We have changed the wording to enriched and depleted in the revised manuscript.

Comment 10

Figure S2 is presented as a box plot even though it is a distribution of only 5 points. I think it would be more transparent to simply show the five points. In my view, a boxplot is appropriate for sample sizes that are large enough to break into quartiles in a meaningful way.

Response 10
Thank you for your comment. We agree and have amended the plot to show the individual data points.

Comment 11
Line 312. The authors use the term "basal angiosperm." I know that this usage is fairly common in the literature, but I would argue that it's misleading when referring to extant taxa that are not actually basal in the tree (see Crisp and Cook 2005 for discussion of this issue; doi: 10.1016/j.tree.2004.11.010). I would suggest "... as they are the sister lineage to rest of the sampled angiosperms in the dataset."

Response 11
We apologise for the imprecise language here. We fully agree and have changed the wording to the reviewer’s suggestion.

Dear Dr. Steven Kelly:

Thank you for your thorough and well considered responses to concerns and suggestions provided by reviewers and editorial board members. We are pleased to inform you that your paper entitled "Widespread adaptive evolution in the photosystems of angiosperms provides insight into the evolution of photosystem II repair." has been accepted for publication in The Plant Cell, pending a final editorial review by a science editor. At this stage, your manuscript will be evaluated by a science editor with respect to its presentation of scientific content, compliance with journal policies, and presentation for a broad readership. The Plant Cell has contracted with Plant Editors (planteditors.com) to provide this service to our authors, and you will soon receive additional information on this process.

Please note that each author needs to link their ORCID identifier to their account in the system before your manuscript can be published. If any authors do not have an ORCID linked to their account, they will receive a message with a link to complete this task. Please ensure that ALL of your coauthors have completed this task as soon as possible.

LICENSE INFORMATION Shortly after your paper is sent to production, you will receive an email from our publisher's author support team, **SciPris**, with information on article processing charges, as well as the types of Open Access license you would be able to purchase. The accepted version of your manuscript will not appear on our Advance Articles page until the license is selected and signed, so please look out for an email from SciPris. For more information see the Article Publication Fees section of our author guidelines: https://academic.oup.com/plcell/pages/General-Instructions

The Plant Cell and The Arabidopsis Information Resource (TAIR) are collaborating to collect functional annotation data about Arabidopsis genes from authors. This includes information about the gene's molecular function (e.g., kinase activity, ATP synthetase activity), the biological process/es it is involved in (e.g., endosperm development, threonine biosynthesis), its subcellular location (e.g., nucleus, ER), anatomical or developmental expression pattern (e.g., leaf, ovule, flower stage 10, seedling stage), or its partner in a protein-protein interaction (e.g., AT1G01010 interacts with AT1G01020).

If your paper contains results falling into one or more of these categories for Arabidopsis genes, we request that you now submit these data for inclusion in TAIR by filling in the form provided at the following URL: https://www.arabidopsis.org/doc/submit/functional_annotation/123. If you need further clarification on what types of data can be submitted please contact curator@arabidopsis.org.

Finally, we encourage your submission of artwork for the journal cover. If there is an image or illustration related to your paper that you would like to have considered, please email it in .pdf or .tif format to clowe@aspb.org. An image title, brief, 2-3 sentence description of the image, and the name of the person credited for the image are also encouraged. If your image is selected to appear on the online cover of the journal, you will be contacted with the scheduled issue date and further information.

We look forward to seeing your paper published.

Sincerely,

The Plant Cell Board of Editors ----------------

=====
IMPORTANT REMINDER: PEER REVIEW REPORTS
=====

If you opted to publish a peer review report along with your article during the original submission process, it will be prepared by the editorial staff and publicly posted with your manuscript, inside the zip file that contains any other supplemental material. As a reminder, the peer review report is a public record of all comments from editors and reviewers, as well as your prior responses, as you received them in the decision letters for each draft of your manuscript. If you agreed to publish this report and have changed your mind, or are not sure if you selected this option, please contact the editorial office as soon as possible before signing the license agreement from our publisher.

=====

----FOR ASPB OFFICE USE ONLY (DO NOT EDIT)----
MSID: 40051
Corresponding Author: Prof. Kelly
